# Novel metabolic subtypes in IDH-mutant gliomas: implications for prognosis and therapy

**DOI:** 10.1186/s12885-025-14176-y

**Published:** 2025-04-30

**Authors:** Peng Wang, Jiayi Wang, Zheng Fang, Qiaodong Chen, Ying Zhang, Xiaoguang Qiu, Zhaoshi Bao

**Affiliations:** 1 Department of Neurosurgery, Beijing Tiantan Hospital, Capital Medical University, Beijing, China; 2 Department of Molecular Neuropathology, Beijing Neurosurgical Institute, Capital Medical University, Beijing, China; 3 Department of Radiation Oncology, Beijing Tiantan Hospital, Capital Medical University, Beijing, China

**Keywords:** Glioma, IDH-mutant, Metabolism, Transcriptome, Gene-expression signature

## Abstract

**Background:**

Although IDH-mutant glioma generally has a better prognosis than their IDH-wildtype counterparts, considerable prognostic heterogeneity persists among patients with the same IDH mutation. Current study has primarily focused on the different IDH statuses or grades, while the metabolic heterogeneity within IDH-mutant gliomas remains insufficiently characterized. This study aims to identify transcriptomic metabolic subtypes and associated immune microenvironment differences to better understand survival variability and potential therapeutic targets in IDH-mutant glioma.

**Methods:**

Patients with IDH-mutant gliomas were included from four public datasets (TCGA, *n* = 373; CGGA325, *n* = 167; CGGA693, *n* = 333; GLASS, *n* = 100), supplemented by 22 cases from Beijing Tiantan Hospital as an independent cohort. Consensus clustering was used to define novel metabolic subtypes. Clinical features were assessed using chi-square tests and Kaplan–Meier analysis. Metabolic profiles were characterized through enrichment analysis and GSVA; immune infiltration was analyzed using CIBERSORTx and ESTIMATE. Tumor samples from the independent cohort underwent untargeted metabolomics for validation. LASSO regression was applied to select metabolic signatures, and the CGP2014 drug library was used for drug screening.

**Results:**

Three metabolic subtypes (C1–C3) with distinct prognoses (*p* < 0.05) were identified. C1 exhibited enhanced carbohydrate and nucleotide metabolism; C2 displayed upregulated amino acid and lipid metabolism; and C3 demonstrated elevated lipid, nucleotide, and vitamin metabolism. These patterns were validated in the independent cohort. Subtypes were also correlated with immune infiltration. A 13-gene metabolic signature was established to stratify prognostic risk and suggest subtype-specific drug sensitivities.

**Conclusions:**

Our study provided a novel metabolic subtype for IDH-mutant glioma and highlighted these patients' metabolic heterogeneity and potential therapeutic strategies.

**Supplementary Information:**

The online version contains supplementary material available at 10.1186/s12885-025-14176-y.

## Background

The latest World Health Organization (WHO) classification of central nervous system tumors has significantly refined glioma taxonomy by integrating molecular markers with traditional histopathology [1]. Among these, isocitrate dehydrogenase (IDH) mutation status has emerged as a crucial diagnostic criterion that profoundly distinguishes the biological behavior and clinical outcomes of gliomas [2, 3]. IDH-mutant gliomas generally exhibit more favorable prognoses than their IDH wild-type counterparts [4, 5]. However, increasing evidence suggests that considerable metabolic heterogeneity within IDH-mutant tumors themselves potentially influences treatment response and survival [6, 7].

Metabolic reprogramming is now recognized as a hallmark of gliomas, contributing to tumor growth, therapeutic resistance, and disease progression [8–10]. Specifically, IDH mutations, which frequently occur in lower-grade and secondary high-grade gliomas, induce distinct metabolic alterations, such as the accumulation of the oncometabolite 2-hydroxyglutarate (2-HG), that substantially affect tumor growth, cellular invasion, and sensitivity to therapies [11–13]. Despite the well-established findings of different IDH status and different grades, the metabolic landscape of IDH-mutant gliomas remains incompletely understood, impeding the development of targeted therapies. [14–16].

Identifying molecularly distinct metabolic subtypes within IDH-mutant gliomas is, therefore, essential for developing more targeted and effective treatments. Metabolic subtyping may unveil unique vulnerabilities that can be exploited therapeutically, enabling a more personalized approach to care. Moreover, understanding these distinct metabolic phenotypes may help clinicians more accurately anticipate disease progression and therapeutic response [17, 18].

In this study, we performed bioinformatics analysis on metabolic-associated RNA sequencing data from IDH-mutant gliomas, revealing three distinct metabolic subtypes. We characterized these subtypes according to their metabolic characteristics, associated gene expression patterns, and potential therapeutic implications. Our findings provide novel insights into the complex metabolic heterogeneity inherent in IDH-mutant gliomas and establish a foundation for more personalized therapeutic approaches.

## Methods

### Patients and datasets

A total of 973 patients were recruited from four datasets (Table S1): The Cancer Genome Atlas (TCGA, *n* = 373, http://cancergenome.nih.gov/), Chinese Glioma Genome Atlas (CGGA 325, *n* = 167; CGGA 693, *n* = 333, http://www.cgga.org.cn), and The Glioma Longitudinal AnalySiS (GLASS, *n* = 100, https://glass-consortium.org/) [19–22]. Inclusion criteria were: (1) confirmed IDH-mutant status, (2) availability of complete RNA-seq sequencing data, and (3) complete follow-up information. Exclusion criteria included (1) IDH-wildtype or unknown mutation status and (2) missing follow-up or molecular pathological data. Tumor grade and primary/recurrent status were not considered exclusion criteria. For all eligible patients, RNA-seq data, clinical parameters, and molecular pathological information were collected, including age, sex, histopathological classification, survival data, Telomerase reverse transcriptase promoter (TERTp) mutation status, MGMT promoter methylation, 1p/19q co-deletion status, Verhaak subtype [24], primary/recurrent status (PRS), and IDH mutation status [25]. Additionally, 22 IDH-mutant glioma patients from Beijing Tiantan Hospital were collected for LC/MS tests. The study protocol adhered to the Declaration of Helsinki and was approved by the Ethics Committee of Beijing Tiantan Hospital (KY 2020–093 - 02). All patients provided written informed consent before data acquisition.

### Identification of novel metabolic subtypes

The TCGA dataset was designated as the discovery cohort, while CGGA 325, CGGA 693 and GLASS were served as independent validation cohorts. A curated list of 2,752 metabolism-related genes from prior studies (Table S1) was used as the basis for analysis [26, 27]. Cox regression analysis was performed, and 351 overall survival-related genes were selected in the TCGA dataset for further consensus clustering analysis. Then, the candidate genes with high median absolute deviation (MAD) values were selected for consensus clustering to identify new subtypes. The cumulative distribution function (CDF) and consensus heatmap were adopted to assess the optimal K [28]. Spearman correlation analysis was used to score each patient in CGGA325, CGGA693 and GLASS to validate the subtypes in the other datasets. Then, we annotated each patient in the CGGA 325, CGGA 693, and GLASS datasets to the novel metabolic subtype according to the relevance score.

### Pathways analysis associated with novel metabolic subtypes

We identified 114 metabolic pathways from previously published articles and performed Gene set variation analysis (GSVA) to score each patient (R package “GSVA package v1.42”) [27, 29]. Prior to GSVA, one-way analysis of variance (ANOVA) was conducted to identify significantly variable pathways across subtypes. Based on pathway scores, supervised clustering and visualization were performed using the “ComplexHeatmap” R package (v2.10).

### Selection of metabolism-related gene signatures

The Least Absolute Shrinkage and Selection Operator (LASSO) regression algorithm was applied to the 351 survival-associated genes to identify prognostically relevant metabolic genes. Using the one-standard-error (λ-se) criterion, we selected 13 variables with non-zero regression coefficients for inclusion in subsequent analysis models. The coefficients of the 13 genes identified by LASSO regression were used as weights and multiplied by each patient's corresponding gene expression levels to calculate a risk score. This score was then included as a variable in the survival model. Patients were stratified into high- and low-risk groups based on the optimal cut point. Additionally, we used the CGP2014 drug library to predict drug sensitivity for the signature.

### Bioinformatics and statistical analyses

Bioinformatics analyses were carried out utilizing RStudio (version: 4.1.2). Gene Ontology (G-O) analysis and Kyoto Encyclopedia of Genes and Genomes (KEGG) analyses were conducted via the DAVID online platform (https://david.ncifcrf.gov/) [30]. Differentially expressed genes (DEGs) were identified using the limma package (thresholds, *p* ≤ 0.05 and |log₂FC|> 1) to determine upregulated and downregulated genes across metabolic subtypes in each dataset. Genes with the same trend in ≥ 3 datasets (C1 and C2) or ≥ 2 datasets (C3) were retained. These DEGs were used in the G-O and KEGG analyses, and subsequent results were visualized by the R package “clusterProfiler” (v4.2.2) [31]. Principal Component Analysis (PCA) was performed employing the R package “FactoMineR” (v2.8) and enhanced with the R package “Factoextra” (v1.0.7). Survival analysis and the drafting of Kaplan–Meier curves were executed by the R package “Survival” (v3.5–5), with Spearman's correlation analysis completed through the inbuilt functions of RStudio. Consensus clustering analysis was conducted with the R package “ConsensusClusterPlus” (v1.58) [32]. The “CIBERSORT.R” script conducted CIBERSORT analysis, and the Estimation of Stromal and Immune cell populations in Malignant Tumors using Expression data (ESTIMATE) analysis was concluded utilizing the R package “estimate” (v1.0.13) [33, 34].

All statistical analyses were conducted in R and SPSS 26.0 (IBM, Chicago, IL, USA). Survival differences were evaluated using Kaplan–Meier and log-rank tests. Chi-square and Fisher’s exact tests were used for comparisons of categorical variables. One-way ANOVA was used for comparisons among three groups. Univariate and multivariate Cox regression models were applied to determine prognostic factors. A two-tailed *p*-value ≤ 0.05 was considered statistically significant.

### Untargeted metabolomic profiling of gliomas using high-resolution LC/MS

Untargeted LC − MS/MS metabolomics was conducted using a Nexera XR LC- 20 ADXR system (Shimadzu Europa, Duisburg, Germany) coupled to an AB SCIEX TripleTOF 5600 + system (AB SCIEX, Foster City, USA). Separation was achieved using a Waters ACQUITY HSS T3 column (2.1 × 100 mm, 1.8 μm; Waters, Milford, MA, USA). Reagent grades for metabolomic analysis were purchased from Fisher Chemicals (Marshalltown, USA). The injection volume was 3 μl for positive ESI mode and 5 μL for negative ESI mode. For both ion modes, the column temperature was maintained at 35 °C. A gradient program was used at a flow rate of 250 μL/min; mobile phase A was water containing 0.1% formic acid and mobile phase B was pure acetonitrile. The gradient program was applied as follows: 98% A/2% B for 0–0.1 min; increased to 40% A/60% B from 0.1 to 5 min; maintained at 40% A/60% B from 5 to 10 min; increased to 0% A/100% B from 10 to 17 min; maintained at 0% A/100% B from 17 to 20 min; decreased to 98% A/2% B from 20 to 20.1 min; The stop time was 0.1 min. For the acquisition of the MS/MS data, the MS system was operated using an AB SCIEX TripleTOF 5600 mass analyzer with information dependent acquisition (IDA) for ions in both positive and negative modes. For positive mode, the collision energy was set to 10 V, declustering potential was set to 80 V, and ion spray voltage was set to + 5500 V with mass range from 100 to 1000 m/z. For negative mode, the collision energy was set to − 10 V, declustering potential was set to − 80 V, and ion spray voltage was set to − 4500 V with a mass range from 100 to 1000 m/z. The other source parameters were as follows: the ion source gas 1 was set at 50 psi, the ion source gas 2 was set at 55 psi, the curtain gas was set at 35 psi, and the drying temperature was set at 450 °C. The instrument was mass calibrated using Calibration Solution for AB SCIEX TripleTOF 5600 (part no. 4460131) for ESI positive mode and Calibration Solution for AB SCIEX TripleTOF 5600 (part no. 4460134) for ESI negative mode [35].

## Results

### Consensus clustering identifies three novel metabolic subtypes in IDH-mutant glioma

**Fig. 1:**
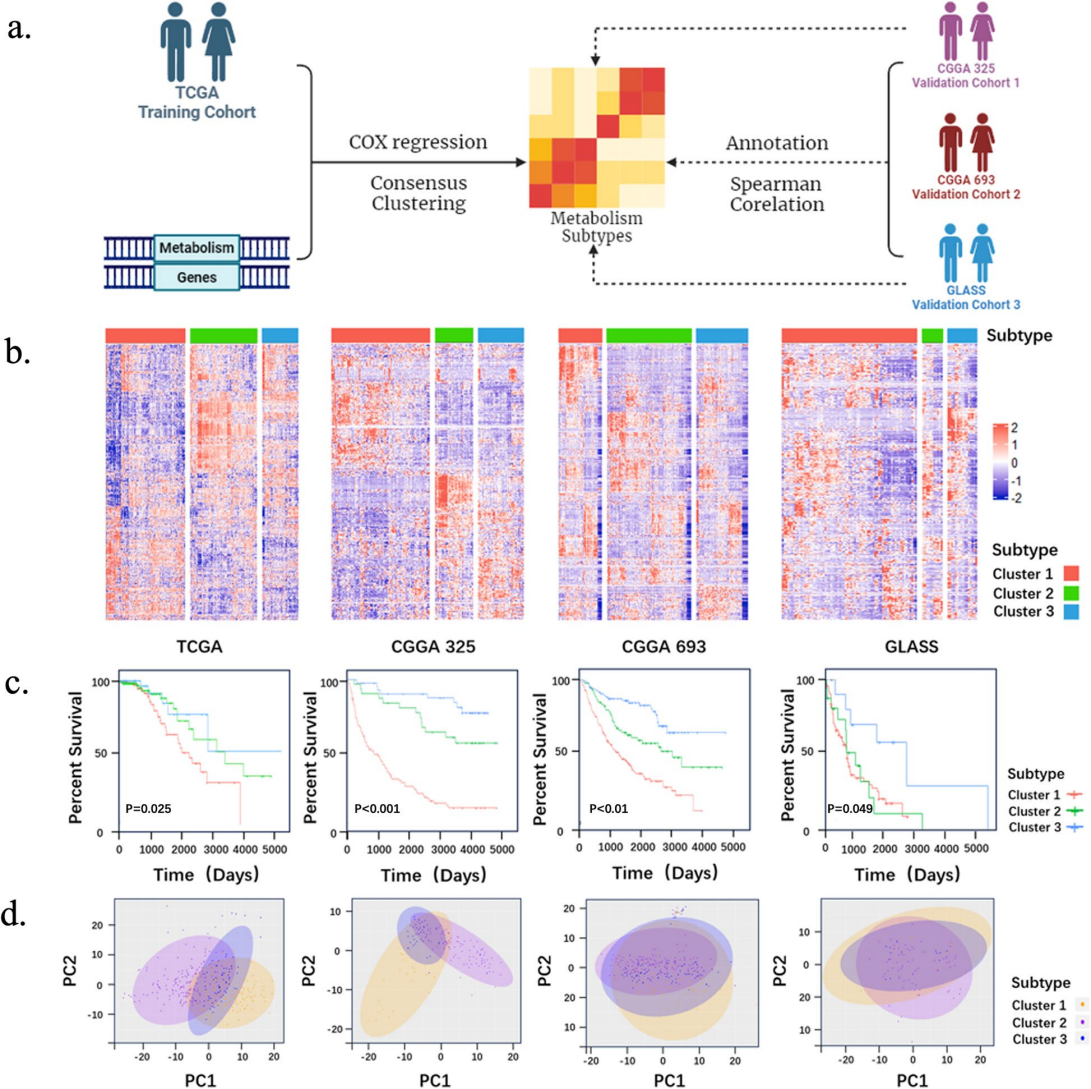
Identification of novel metabolic subtypes in IDH-mutant glioma through metabolism gene profiling. **a**. The flowchart and methodologies adopted throughout the study are shown, with TCGA as the discovery set, and CGGA and GLASS as validation sets; **b**. Heatmaps of three metabolic subtypes defined in four cohorts; **c**. Survival analyses show significant differences between the novel metabolic subtypes in four LGG cohorts. *P* value was calculated by the log-rank test among subtypes; **d**. Principal component analysis (PCA) of three metabolic subtypes using candidate genes

In the discovery cohort (TCGA, *n* = 373), univariate Cox regression identified 351 metabolism-related genes significantly associated with overall survival. Consensus clustering based on these genes revealed three distinct metabolic subtypes, designated as cluster 1 (C1), cluster 2 (C2), and cluster 3 (C3) (Figure S1). Subtypes in the validation cohorts (CGGA325, CGGA693, GLASS) were assigned by calculating Spearman correlation coefficients between individual transcriptomic profiles and subtype centroids from the TCGA cohort (Fig. 1a).

### Prognostic and clinical characteristics of the three metabolic subtypes

Expression heatmaps of the 351 metabolic genes demonstrated clear differences in expression patterns across the three subtypes (Fig. 1b). Survival analysis showed significant differences in prognosis among the subtypes in both the discovery (*p* = 0.025) and validation datasets. Patients in C1 subtype always had the worst prognosis and C3 with the most favorable outcome (Fig. 1c, CGGA 325: *p* < 0.01; CGGA 693: *p* < 0.01; GLASS: *p* = 0.049). Pairwise comparisons revealed that in TCGA, C2 and C3 were not significantly different (*p* = 0.53), while in GLASS, C1 and C2 were not well-separated (*p* = 0.92). Nonetheless, all subtype comparisons were statistically significant in at least one cohort (Figure S2). PCA analysis demonstrated a clear separation of subtypes in TCGA, CGGA 325, and CGGA 693 but not in the GLASS dataset (Fig. 1d).

**Fig. 2:**
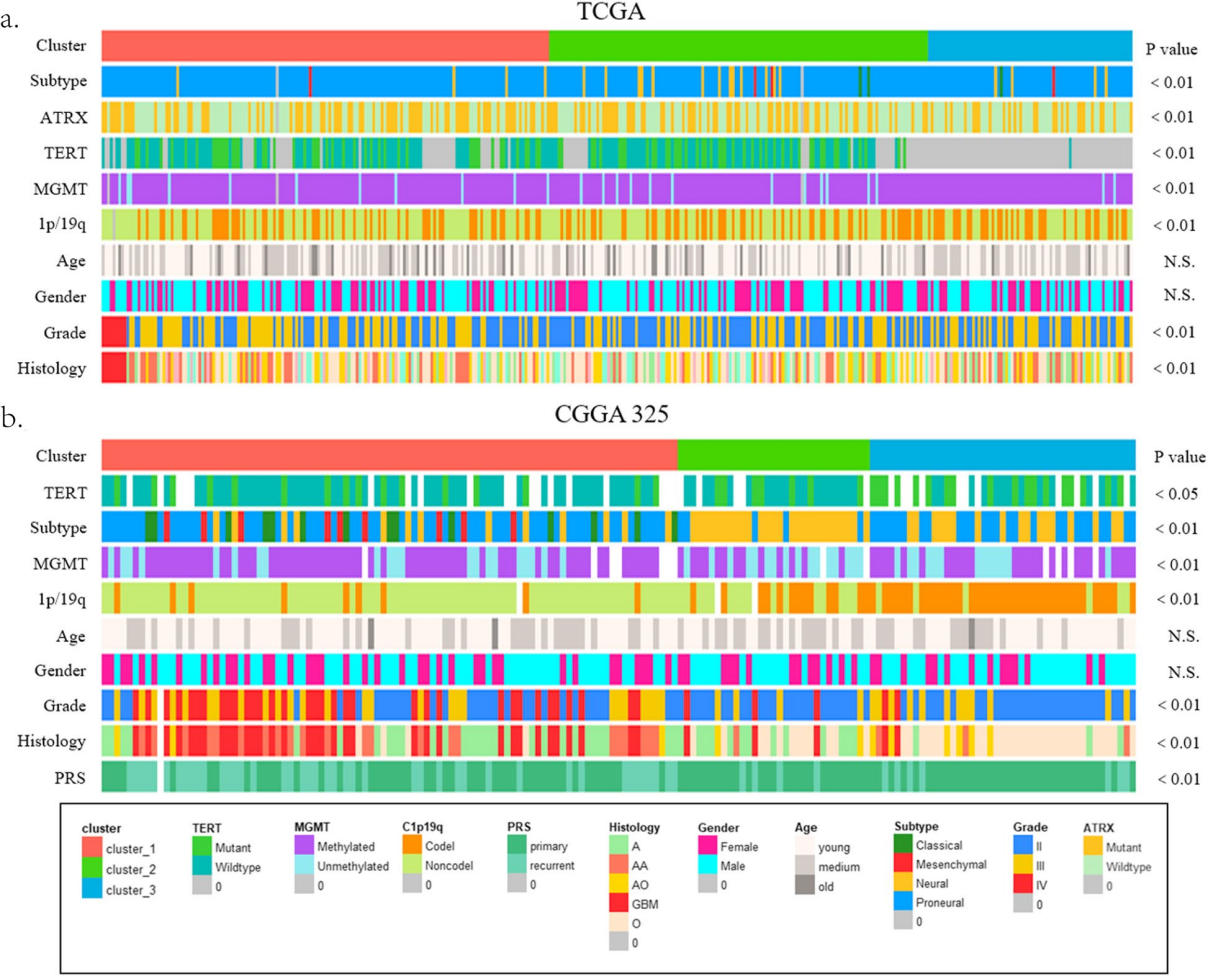
Clinical characteristics of metabolic subtypes in TCGA and CGGA- 325 cohorts. **a**. Clinical characteristics in the TCGA cohort; **b**. Clinical characteristics in the CGGA- 325 cohort. The chi-square test was used for statistical analysis. 0 means not applicable

Clinical and molecular characteristics were also subtype-specific (Fig. 2, Figure S3). C1 was enriched for glioblastoma and anaplastic astrocytoma, whereas oligodendroglioma was more common in C3, and astrocytoma/anaplastic oligodendroglioma predominated in C2 (*p* < 0.01). High-grade glioma tends to appear in C1 (*p* < 0.01). Molecular subtype analysis revealed that C1 was primarily Proneural and Classical; C2 was predominantly Neural; and C3 had mixed features (*p* < 0.01). All Mesenchymal occurred in C1. Furthermore, 1p/19q co-deletion and TERT promoter mutations were significantly more common in C3 and C1, respectively (*p* < 0.01). PRS status differed significantly only in CGGA 325 (*p* < 0.01). Age distribution did not differ significantly across subtypes in any dataset (Table S2–S6).

### Function and pathway characterization of metabolic subtypes

**Fig. 3:**
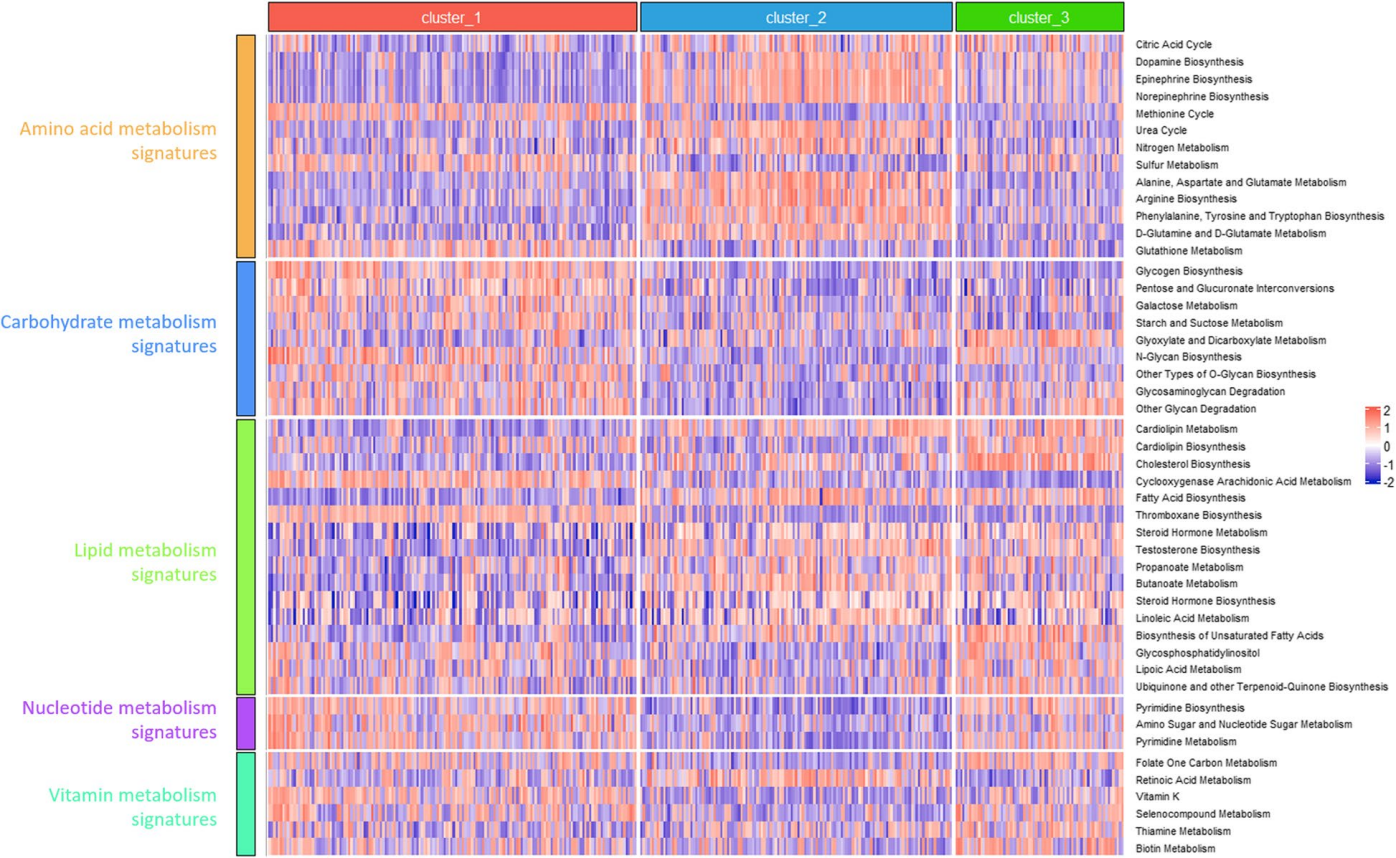
Association between metabolism-relevant signatures and novel metabolic subtypes. Heatmaps of differential enrichments of metabolism-related signatures in the TCGA cohort. Amino acid, carbohydrate, lipid, nucleotide, and vitamin metabolism signatures were presented. The statistical difference was compared through the ANOVA test, and the *P* value≤0.05 was considered as significant

Out of 114 curated metabolic pathways, 47 were significantly different among subtypes based on ANOVA across all datasets (Table S7). These were categorized into five groups: amino acid metabolism (13 pathways), carbohydrate metabolism (9), lipid metabolism (16), nucleotide metabolism (3), and vitamin metabolism (9) [36]. Through clustering analysis in the discovery set, we found that in C1 subtype, Carbohydrate metabolism and Nucleotide metabolism were upregulated, while Amino acid metabolism and Lipid metabolism were downregulated. In C2 subtype, Amino acid and Lipid metabolism were upregulated, while Carbohydrate and Nucleotide metabolism were downregulated. In C3 subtype, functions related to Lipid metabolism, Nucleotide metabolism, and Vitamin metabolism were upregulated. In contrast, Amino acid metabolism and Carbohydrate metabolism were downregulated (Fig. 3). And similar findings were observed in three validation sets (Figure S4-S6).

**Fig. 4:**
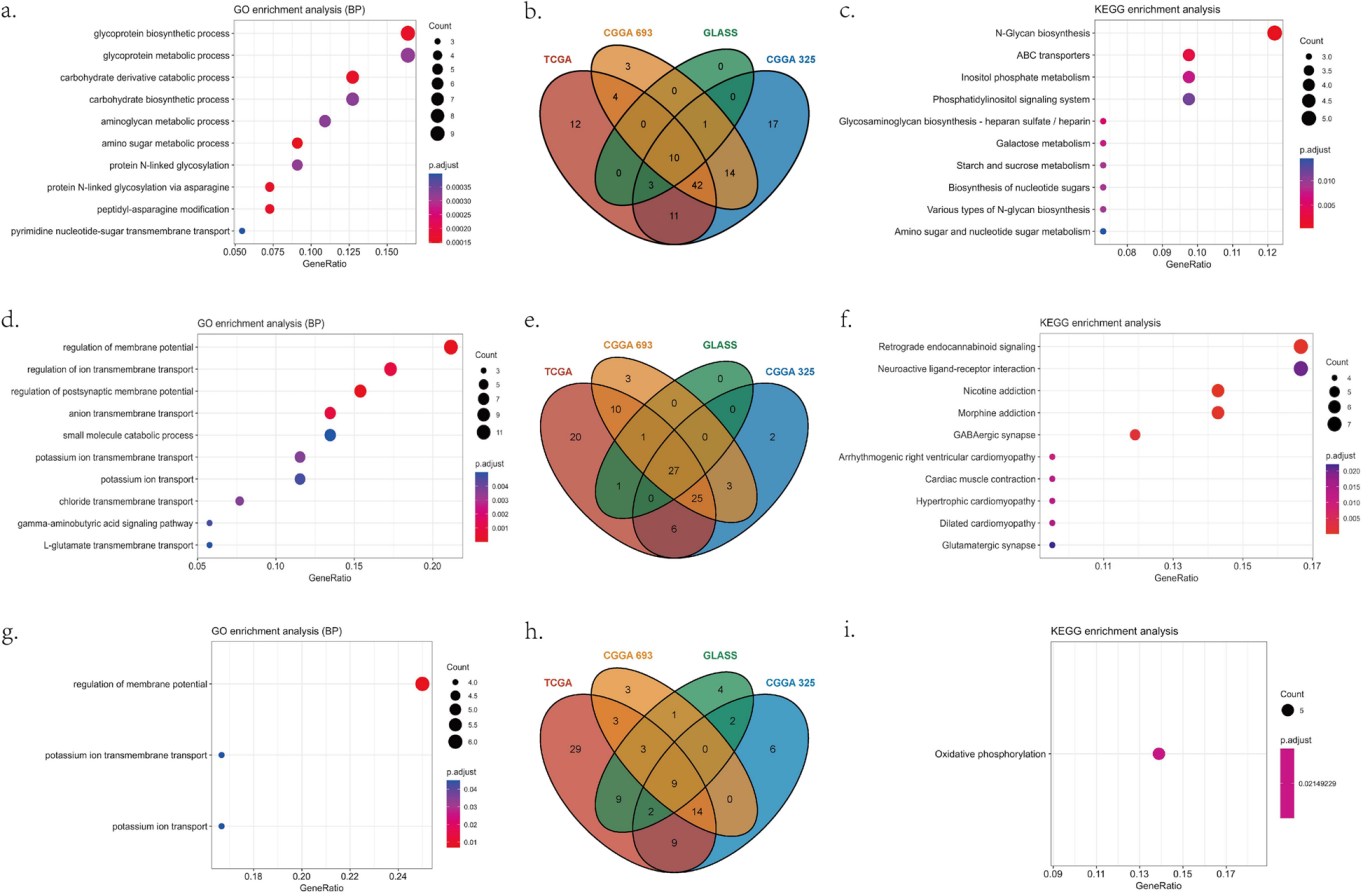
G-O and KEGG pathways up-regulated in C1, C2, C3 subtype representatively. **a**. up-regulated GOBP functions in C1 subtype; **b**. up-regulated genes in C1 subtype; **c**. up-regulated KEGG pathway analysis in C1 subtype; **d**. up-regulated GOBP functions in C2 subtype; **e**. up-regulated genes in C2 subtype; **f**. up-regulated KEGG pathway analysis in C3 subtype; **g**. up-regulated GOBP functions in C3 subtype; **h**. up-regulated genes in C3 subtype; **i**. up-regulated KEGG pathway analysis in C3 subtype

**Fig. 5:**
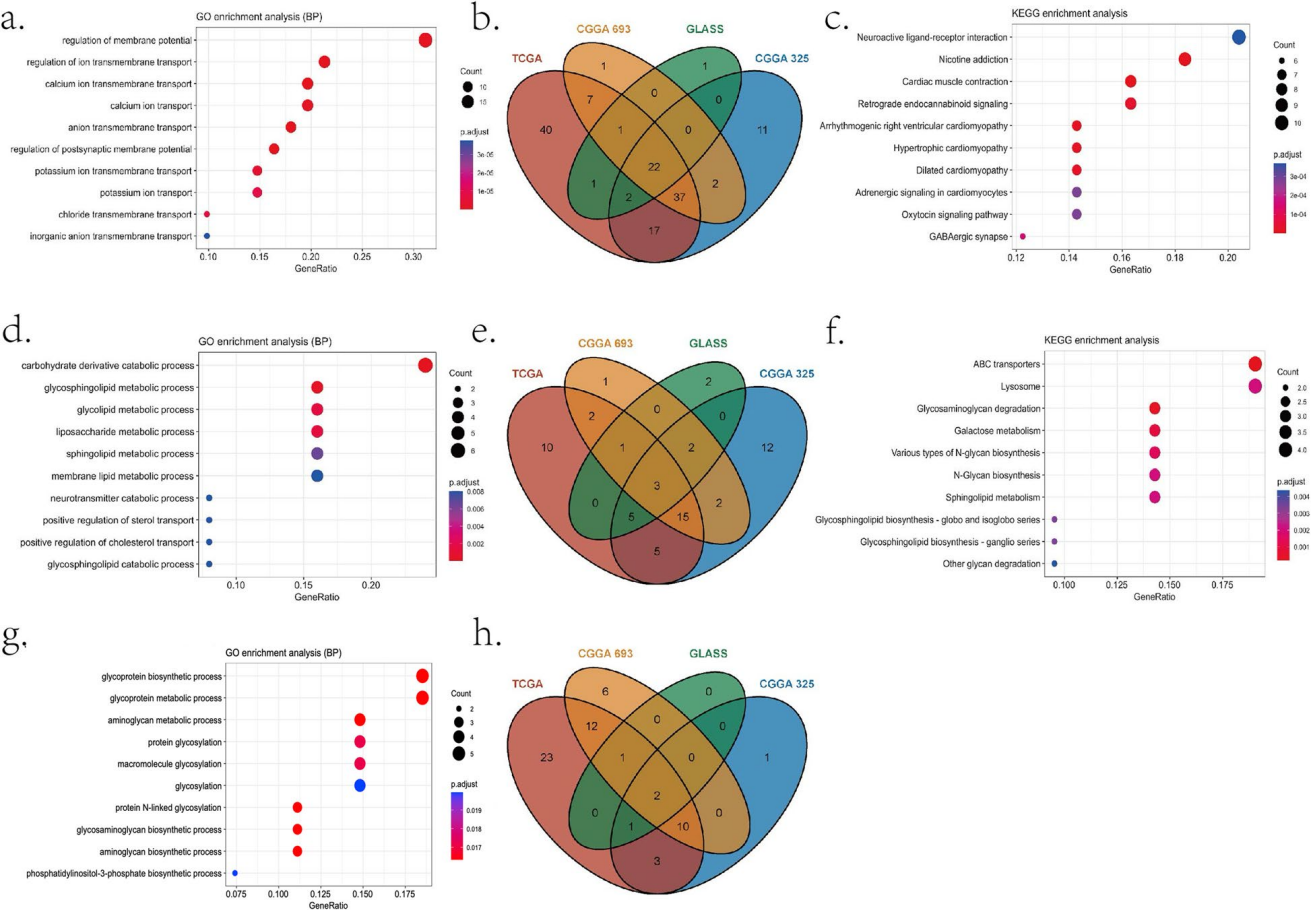
G-O and KEGG pathways down-regulated in C1, C2, C3 subtype representatively. **a**. down-regulated GOBP functions in C1 subtype; **b**. down-regulated genes in C1 subtype; **c**. down-regulated KEGG pathway analysis in C1 subtype; **d**. down-regulated GOBP functions in C2 subtype; **e**. down-regulated genes in C2 subtype; **f**. down-regulated KEGG pathway analysis in C2 subtype; **g**. down-regulated GOBP functions in C3 subtype; **h**. down-regulated genes in C3 subtype

The G-O analysis showed that the upregulated functions in the C1 subtype were mainly related to carbohydrate metabolism and protein synthesis processes (Fig. 4a); in the C2 subtype, the upregulated pathways mainly involved transmembrane ion transport and neurotransmission regulation (Fig. 4d); in C3 subtype the upregulated functions were alcohol and fatty acid metabolic processes (Fig. 4g and Figure S7). While the downregulated functions in the C1 subtype primarily included ion transport and membrane potential regulation (Fig. 5a), in the C2 subtype, the downregulated pathways were mainly associated with lipid metabolism, including the regulation of glycolipid, sphingolipid, and membrane lipid metabolism and transport (Fig. 5d); in the C3 subtype, the downregulated functions were glycoprotein metabolism and glycosylation modification (Fig. 5g and Figure S7).

KEGG pathway analysis showed that C1 had upregulated biosynthetic pathways related to ribonucleotides, N-glycosylation, glycogen synthesis, and sugar transport (Fig. 4c). C2 was enriched in pathways involving neurotransmitter-receptor interactions, addiction-related signaling, and GABAergic/glutamatergic metabolism (Fig. 4f). C3 exhibited upregulation only in oxidative phosphorylation (Fig. 4i). Downregulated pathways in C1: GABA synapses, adrenergic signaling, and neuroactive ligand-receptor interactions (Fig. 5c); C2: carbohydrate metabolism, glycosylation, lysosomal degradation, and lipid metabolism (Fig. 5f). The downregulated pathways in the C3 subtype involved glutathione synthesis, nucleotide synthesis, and extracellular matrix synthesis, but the results were not significant enough due to the small number of genes involved.

### Immune features of the novel metabolic subtypes

**Fig. 6:**
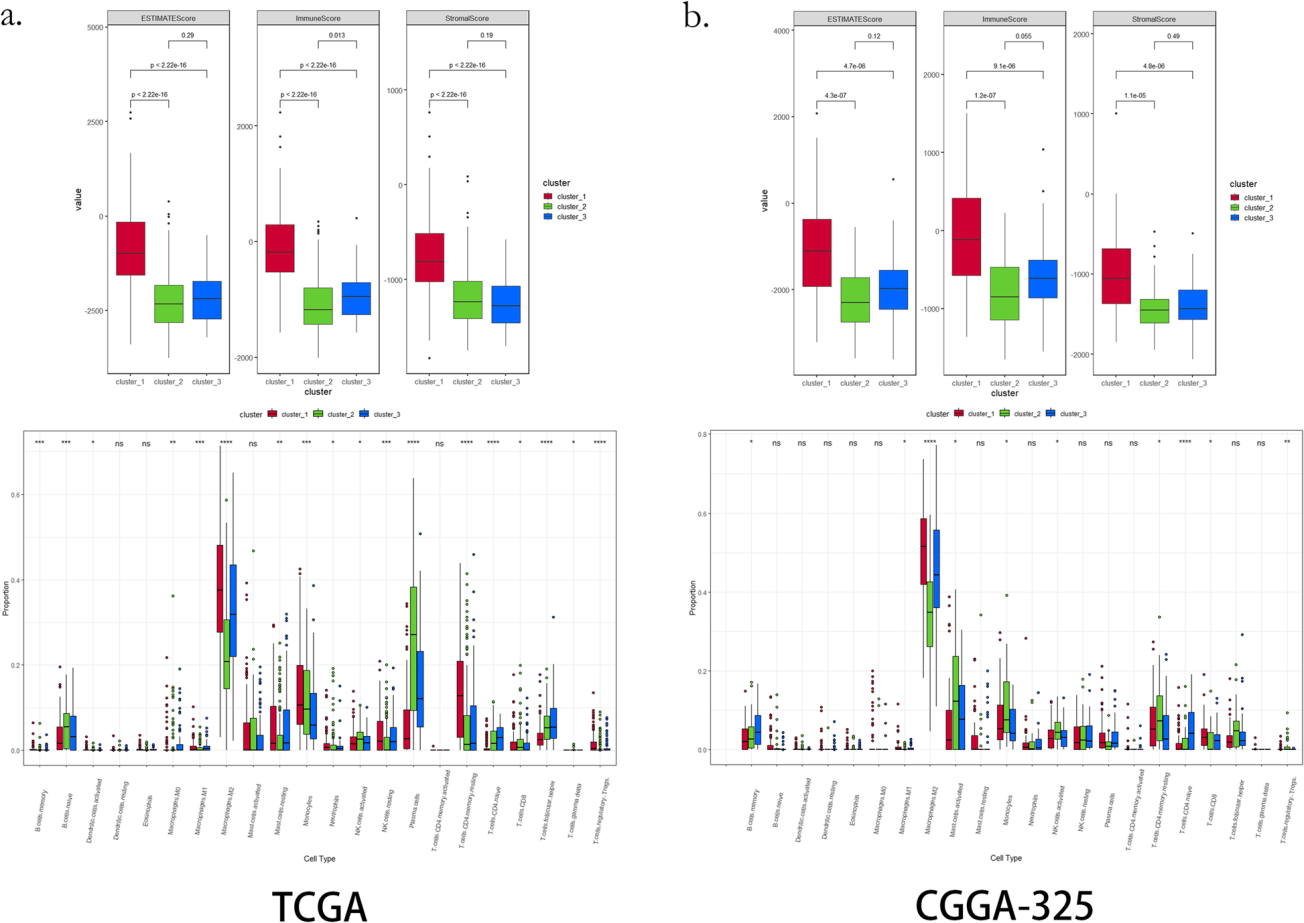
Immune infiltration of three metabolic subtypes in TCGA and CGGA- 325 cohorts. **a**. The ESTIMATE is used to predict immune and stromal scores and CIBERSORT is used to predict the types of immune cells in the TCGA cohort; **b** ESTIMATE is used to predict immune and stromal scores and CIBERSORT is used to predict the types of immune cells in the CGGA- 325 cohort

Through ESTIMATE analysis, we found the ESTIMATE score and Stromal Score in the C1 subtype were significantly higher than the C2 and C3 subtypes (Fig. 6a, C1 vs. C2: *p* < 0.0001, C1 vs. C3: *p* < 0.0001). We also found that the Immune Score in the C1 subtype, which had the worst prognosis, was significantly higher than the other two subtypes (Fig. 6a, C1 vs. C2: *p* < 0.0001, C1 vs. C3: *p* < 0.0001). Similar findings were observed in the three validation sets (Fig. 6b, Fig. S8a & S8b). As for tumor purity, the C1 subtype shows the lowest score in all datasets (*p* < 0.01), which is consistent with the stromal and immune scores (Figure S9). CIBERSORT analysis revealed subtype-specific immune cell distributions. Compared with C3 subtype, which has the best prognosis, C1 showed a decrease in naive B cells (*p* < 0.001), M1 macrophages (*p* < 0.001), NK cells (*p* < 0.01), plasma cells (*p* < 0.0001), naive CD4 cells (*p* < 0.05), helper T cells (*p* < 0.0001); increase in M2 macrophages (*p* < 0.0001), monocytes (*p* < 0.001), CD4 memory cells (*p* < 0.0001), and Tregs (*p* < 0.0001) (Fig. 6a). Similar findings were observed in three validation sets (Fig. 6b & Figure S8).

### Identification and drug sensitivity prediction of a novel metabolic signature

**Fig. 7:**
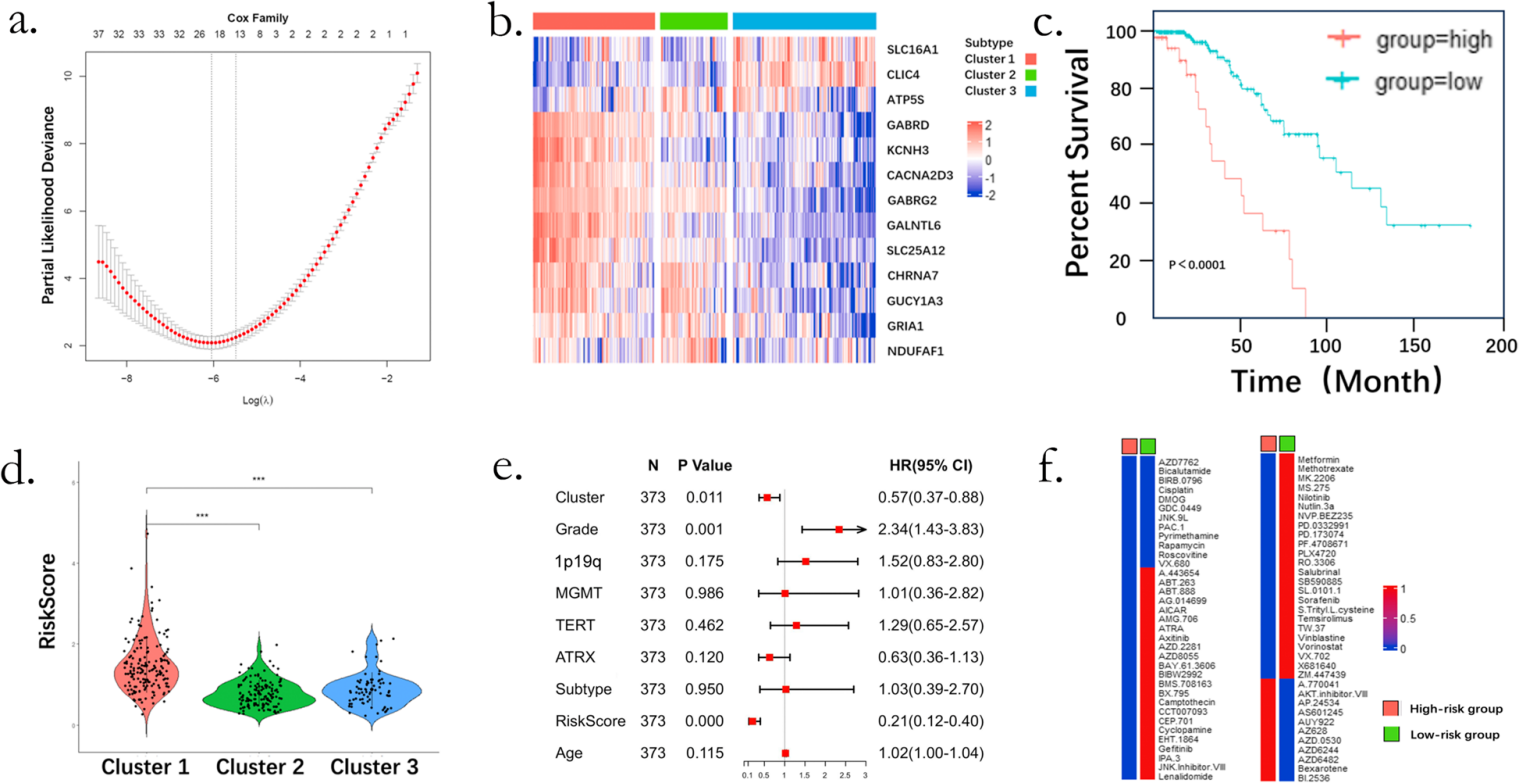
Identification of a metabolic signature and predictive of drug sensitivity. **a**. Cross-validation for tuning parameter selection in the proportional hazards model; **b**. Heatmap showing the expression levels of 13 signature genes; **c**. Kaplan–Meier survival analysis of the metabolic signature in patients of IDH-mutant gliomas. *P* value was calculated by the log-rank test; **d**. Risk score analysis in different metabolic subtypes; **e**. Cox proportional hazards regression forest plot of novel metabolic subtypes compared with other features, “Cluster” means novel metabolic classification, “subtype” means Verhaak’s transcriptional classification; **f**. Drug sensitivity prediction analysis for the top 70 drugs in the CGP2014 drug library

LASSO regression analysis in the TCGA cohort yielded a 13-gene metabolic signature: *SLC25A12, GABRG2, GALNTL6, CACNA2D3, GABRD, KCNH3, CHRNA7, GRIA1, ATP5S, GUCY1A3, NDUFAF1, SLC16A1*, and *CLIC4* (Fig. 7a). These genes showed distinct expression patterns across subtypes (Fig. 7b). The derived risk score effectively stratified patients into high- and low-risk groups with significant survival differences (*p* < 0.0001, Fig. 7c). C1 exhibited significantly higher risk scores compared to C2 and C3 (both *p* < 0.0001, Fig. 7d). Multivariate Cox regression analysis confirmed that in addition to tumor grade, both the metabolic subtypes and the risk score served as independent prognostic factors, even after adjustment for established clinical variables (Fig. 7e). These results were validated in CGGA325, CGGA693, and GLASS (Figure S10).

Drug sensitivity prediction using the CGP2014 library identified 138 candidate compounds. Among them, 104 drugs were predicted to be more effective in the low-risk group, 23 in the high-risk group, and 12 showed no significant difference (Fig. 7f & Figure S11).

### Independent cohorts validate new metabolic profiling features

**Fig. 8:**
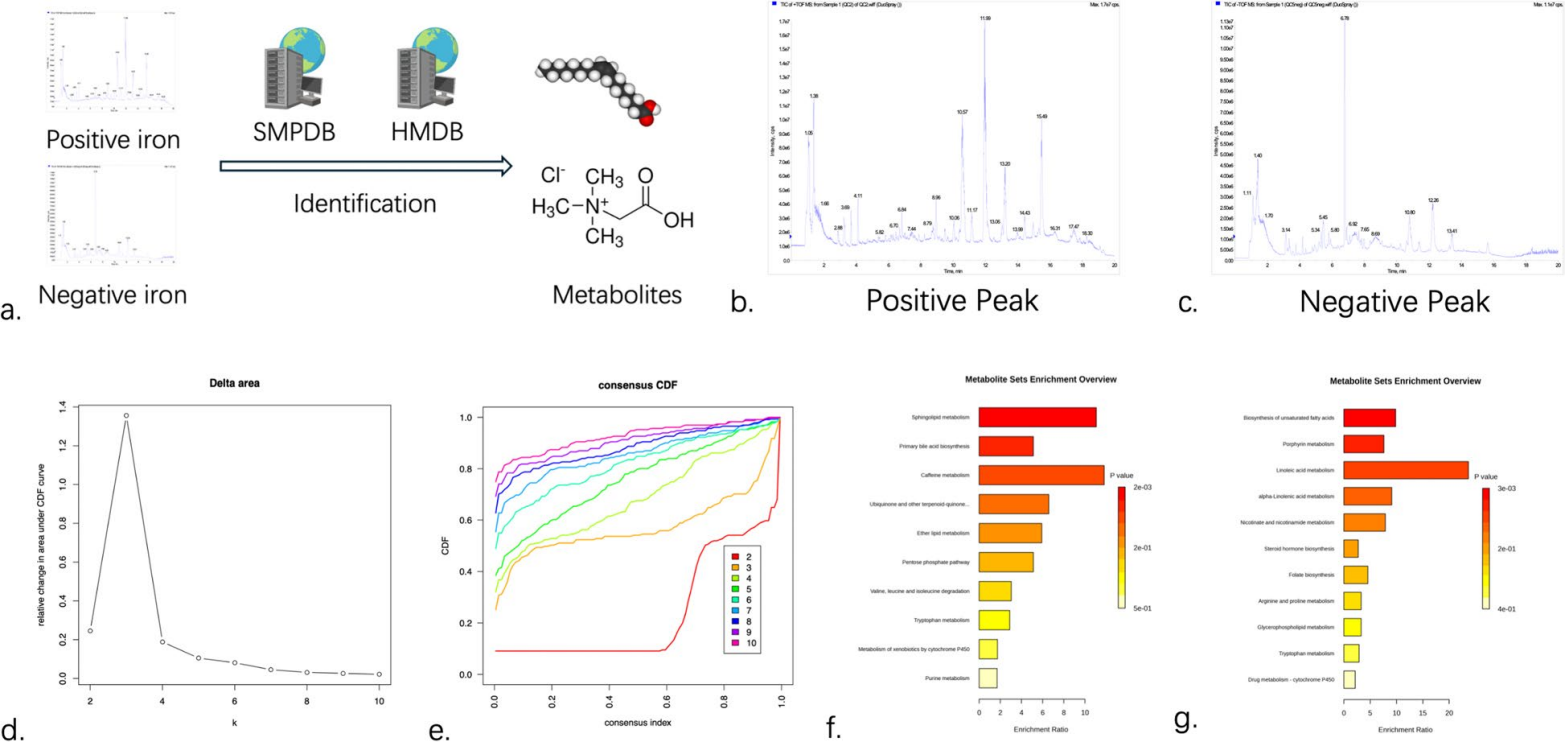
Independent validation cohort validates the metabolic features of IDH-mutant glioma patients. **a**. LC/MC ion identification flowchart; **b**. Statistics of positive ion peak accumulation; **c**. Statistics of negative ion peak accumulation; **d**. Consensus clustering CDF curve results; **e**. Consensus clustering CDF optimal grouping results; **f**. Enrichment analysis of upregulated metabolic pathways in astrocytoma; **g**. Enrichment analysis of upregulated metabolic pathways in oligodendroglioma

We collected blood samples from 22 patients with IDH-mutant gliomas at Beijing Tiantan Hospital, comprising 10 cases of astrocytoma (WHO Grade 2–3), 10 cases of oligodendrogliomas (WHO Grade 2–3), and 2 cases of anaplastic astrocytoma (WHO Grade 4). Untargeted mass spectrometry analysis was performed on these samples (Fig. 8a). A total of 17,363 ions were detected, including 10,146 positive ions (Fig. 8b) and 7,217 negative ions (Fig. 8c). Metabolite identification was performed using the HMDB (Human Metabolome Database) and SMPDB (Small Molecule Pathway Database) databases, resulting in the identification of 1,144 metabolites, which were subsequently used for further analysis (Table S8).

Consensus clustering based on metabolite profiles clearly separated tumors into three distinct groups (Fig. 8d–e). KEGG enrichment analysis of differentially expressed metabolites (|Log_2_FC|> 1) between astrocytomas and oligodendrogliomas revealed significant upregulation of specific metabolic pathways in astrocytomas, including sphingolipid metabolism, primary bile acid biosynthesis, the pentose phosphate pathway, and purine metabolism (Fig. 8f). In contrast, oligodendrogliomas showed significant upregulation in pathways such as unsaturated fatty acid biosynthesis, porphyrin metabolism, nicotinate and nicotinamide metabolism, and arginine and proline metabolism (Fig. 8g). These metabolic trends aligned well with transcriptome-derived subtypes.

## Discussion

Previous studies have identified various glioma subtypes based on transcriptomic and genomic profiling. For example, Matthew et al. explored the characterization of high-grade glioma [37], Ceccarelli et al. revealed distinct molecular subsets and progression pathways in diffuse gliomas using integrated genomic analysis [19]. Fan et al. further explored potential metabolic and immune subtypes in lower-grade gliomas [17, 36]. However, the specific metabolic landscape within IDH-mutant gliomas remains poorly characterized. In this study, we identified three novel transcriptome-defined metabolic subtypes (C1–C3) in IDH-mutant gliomas with distinct clinical, metabolic, and immune profiles. These subtypes were validated across multiple public datasets and in an independent cohort using metabolomics profiling.

Interestingly, our C1 subtype partially overlapped with Fan et al.’s M2 subtype, which is characterized by increased carbohydrate metabolism and poor prognosis in lower-grade gliomas. However, our classification expands upon prior work by demonstrating metabolic subtypes'prognostic and biological relevance across the full spectrum of IDH-mutant gliomas [36]. Moreover, including an intermediate C2 subtype—marked by amino acid and lipid metabolism—offers a more nuanced alternative to previous binary models, such as the dichotomy proposed by Bader et al. [38].

Patients in the C1 subtype exhibited the poorest outcomes, while those in the C3 subtype had the most favorable prognosis, a pattern consistently observed across multiple validation datasets. These prognostic differences were closely aligned with clinical and molecular pathological features. For instance, the C1 subtype was enriched with high-grade gliomas, such as GBM and anaplastic astrocytoma, and was predominantly associated with Proneural and Classical molecular subtypes. Besides, clinical analysis shows it has the highest frequency of TERTp mutation, which is consistent with previous studies [1, 39]. Conversely, the C3 subtype was characterized by a predominance of oligodendrogliomas, 1p/19q co-deletion and MGMT promoter methylation which features associated with better clinical outcomes. The C2 subtype displayed an intermediate profile, with a predominance of astrocytomas and anaplastic oligodendrogliomas, aligning with its intermediate prognosis. The observed enrichment of distinct molecular subtypes, histological grades, and chromosomal alterations within each metabolic subtype underscores the intricate relationship between tumor metabolism and the molecular pathology of IDH-mutant gliomas. These findings suggest that metabolic reprogramming is not merely a byproduct of oncogenesis but a critical determinant of glioma biology and patient outcomes.

Functionally, metabolic enrichment analysis revealed that C1 tumors were highly dependent on glucose metabolism and protein biosynthesis, indicative of high anabolic demand to support rapid tumor proliferation [40–42]. Additionally, downregulation of ion transport and neurotransmission-related pathways in C1 subtype suggests a dedifferentiated and metabolically reprogrammed state [43]. In contrast, C3 tumors showed elevated lipid, nucleotide, and vitamin metabolism, along with upregulated oxidative phosphorylation, pointing to a metabolically efficient and potentially less aggressive phenotype [44–46]. The C2 subtype was marked by active amino acid and lipid metabolism, neuroactive signaling, and neurotransmitter-associated pathways—consistent with its intermediate clinical behavior and partial maintenance of neuronal characteristics.

Metabolic rewiring in gliomas has been shown to influence immune microenvironment [47, 48]. Here, we observed that C1 tumors had the poorest outcomes despite their higher immune and stromal scores. This finding contrasts with previous observations that lower immune infiltration is often linked to tumor progression [49]. CIBERSORT analysis provided further clarity: C1 was enriched in immunosuppressive cell populations such as M2 macrophages, monocytes, and regulatory T cells (Tregs), while displaying lower levels of immune effectors like NK cells, naïve B cells, and helper T cells. This composition suggests an immunosuppressive microenvironment that may boost tumor progression and immune evasion. In contrast, the C3 subtype, which demonstrated the most favorable prognosis, was associated with lower immune scores and a less immunosuppressive environment. The reduced immune infiltration in C3 potentially reflect a less aggressive tumor phenotype with diminished immune evasion mechanisms. The intermediate immune profile of the C2 subtype further supports its transitional characteristics between the aggressive C1 and indolent C3 subtypes [50–53].

Through LASSO regression analysis, we identified a 13 metabolic gene signature that stratified patients into high and low-risk groups with distinct prognostic outcomes. The higher risk scores observed in the C1 subtype compared to C2 and C3 further validated the prognostic relevance of this signature. While tumor grade remains a strong predictor of survival, as reflected in our multivariate analysis, our findings demonstrate that the proposed metabolic subtypes provide additional and independent prognostic insight. Rather than replacing established clinical markers, our subtype framework complements existing models and introduces a metabolically informed stratification that may better guide personalized therapeutic decisions. Functional annotation of the signature genes revealed their involvement in diverse metabolic processes, including energy production, neurotransmission, and membrane dynamics, underscoring their centrality to glioma biology [54–57].

Clinical therapies targeting metabolism and immunity are rapidly expanding, our subtype framework provides timely insights. For example, IDH mutation inhibitors such as vorasidenib and immunotherapies including CAR-T cell therapy have shown promise in clinical trials [58–60]. Our findings may guide personalized treatment strategies by aligning metabolic profiles with predicted therapeutic vulnerabilities. Drug sensitivity analysis revealed that high-risk patients (mainly in C1) might benefit from agents targeting carbohydrate and nucleotide metabolism, whereas low-risk patients (mainly in C3) could be more responsive to oxidative phosphorylation or lipid metabolism inhibitors. These findings offer potential intervention targets and provide a basis for future in vitro and in vivo experimental validation.

While our study comprehensively characterizes metabolic subtypes in IDH-mutant gliomas, some limitations remain. Most metabolic and immune inferences were based on transcriptomic data, and the sample size for untargeted metabolomics was limited. Future work should incorporate multi-omics approaches—including proteomics and targeted metabolomics—to refine subtype classification and elucidate underlying mechanisms.

## Conclusions

Our study characterizes three novel metabolic subtypes of IDH-mutant gliomas at the transcriptome level each with distinct prognostic, metabolic, and immune profiles. The 13-gene metabolic signature developed in this study may serve as a potential tool for risk stratification and treatment selection. These findings enhanced our understanding of IDH-mutant glioma; drug screening based on metabolically associated signatures provides potential targets for future treatments.

## Supplementary Information

Supplementary Material 1: Figure S1. Consistent cluster classification diagram. (a). Consensus matrix for k = 2 to k = 10; (b). consensus CDF curve for k = 2 to k = 10; (c). Relative change in area under CDF curve for k = 2 to k = 10; (d). Tracking plot for k = 2 to k = 10.

Supplementary Material 2: Figure S2. The pairwise survival differences analysis in the discovery set and validation sets. (a)-(c). TCGA OS analysis; (d)-(f). CGGA 325 OS analysis; (g)-(i). CGGA 693 OS analysis; (j)-(l). GLASS OS analysis.

Supplementary Material 3: Figure S3. Clinical characteristics of metabolic subtypes in CGGA- 693 and GLASS cohorts. (a). Clinical characteristics in the CGGA- 693 cohort; (b). Clinical characteristics in the GLASS cohort. The chi-square test was used for statistical analysis. 0 means not appliable.

Supplementary Material 4: Figure S4. Association between metabolism-relevant signatures and novel metabolic subtypes. Heatmaps of differential enrichments of metabolism-related signatures in the CGGA- 325 cohort. Amino acid, carbohydrate, lipid, nucleotide, and vitamin metabolism signatures were presented. The statistical difference was compared through the ANOVA test, and the *P* value < 0.05 was considered as significant.

Supplementary Material 5: Figure S5. Association between metabolism-relevant signatures and novel metabolic subtypes. Heatmaps of differential enrichments of metabolism-related signatures in the CGGA- 693 cohort. Amino acid, carbohydrate, lipid, nucleotide, and vitamin metabolism signatures were presented. The statistical difference was compared through the ANOVA test, and the *P* value < 0.05 was considered as significant.

Supplementary Material 6: Figure S6. Association between metabolism-relevant signatures and novel metabolic subtypes. Heatmaps of differential enrichments of metabolism-related signatures in the GLASS cohort. Amino acid, carbohydrate, lipid, nucleotide, and vitamin metabolism signatures were presented. The statistical difference was compared through the ANOVA test, and the *P* value < 0.05 was considered as significant.

Supplementary Material 7: Figure S7. Functional enrichment analysis of the metabolic subtypes in discovery and validation cohort. (a). MF and CC terms in G-O analysis of genes in cluster 1 up and down regulation; (b). MF and CC terms in G-O analysis of genes in cluster 2 up and down regulation; (c). MF and CC terms in G-O analysis of genes in cluster 3 up and down regulation.

Supplementary Material 8: Figure S8. Immune infiltration of three metabolic subtypes in CGGA- 693 and GLASS cohorts. (a). The ESTIMATE is used to predict immune and stromal scores and CIBERSORT is used to predict the types of immune cells in the CGGA- 693 cohort; (b). The ESTIMATE is used to predict immune and stromal scores and CIBERSORT is used to predict the types of immune cells in the GLASS cohort.

Supplementary Material 9: Figure S9. Tumor purity score calculated according to the ESTIMATE algorithm in the TCGA, CGGA 325, CGGA 693, and GLASS database. (a). Tumor purity score of TCGA set; (b). Tumor purity score of CGGA 325 set; (c). Tumor purity score of CGGA 693 set; (d). Tumor purity score of GLASS set.

Supplementary Material 10: Figure S10. Validation of the obtained metabolic signature in three validation cohorts. (a). Survival analyses of the metabolic signature in IDH-mutant gliomas. *P* value was calculated by the log-rank test; (b). Heatmaps show the signature gene expression of three validation cohorts; (c). Distribution of risk scores in cases stratified by metabolic subtype in three validation cohorts.

Supplementary Material 11: Figure S11. Predation of drug sensitivity for the signature in CGP2014 drug library. Total 127 drugs and the resistance drug has been marked in blue cross.

Supplementary Material 12. Table S1. All metabolic gene symbols.

Supplementary Material 13. Table S2. Clinical features of patients in this study.

Supplementary Material 14. Table S3. Clinical features of patients in TCGA cohort.

Supplementary Material 15. Table S4. Clinical features of patients in CGGA 325 cohort.

Supplementary Material 16. Table S5. Clinical features of patients in CGGA 693 cohort.

Supplementary Material 17. Table S6. Clinical features of patients in GLASS cohort.

Supplementary Material 18. Table S7. The 114 GSVA pathways.

Supplementary Material 19. Table S8. untargeted metabolism results.

## Data Availability

The data were on the Supplementary files.

## Abbreviations

TCGA: The Cancer Genome Atlas
IDH: Isocitrate dehydrogenase
MGMTp: O6-methylguanine-DNA methyltransferase promoter
CGGA: Chinese Glioma Genome Atlas
GLASS: The Glioma Longitudinal AnalySiS
TERTp: Telomerase reverse transcriptase promoter
PRS: Primary/recurrent status
GSVA: Gene set variation analysis
ANOVA: A one-way analysis of variance
LASSO: Least Absolute Shrinkage and Selection Operator
G-O: Gene Ontology
KEGG: Kyoto Encyclopedia of Genes and Genomes
PCA: Principal Component Analysis
ESTIMATE: Estimation of Stromal and Immune cell populations in Malignant Tumors using Expression data
