# Supplementary figures and images for: Novel metabolic subtypes in IDH-mutant gliomas: implications for prognosis and therapy
Source: BMC Cancer. 2025 Apr 30;25:815. doi: 10.1186/s12885-025-14176-y (PMC12044917; doi:10.1186/s12885-025-14176-y)

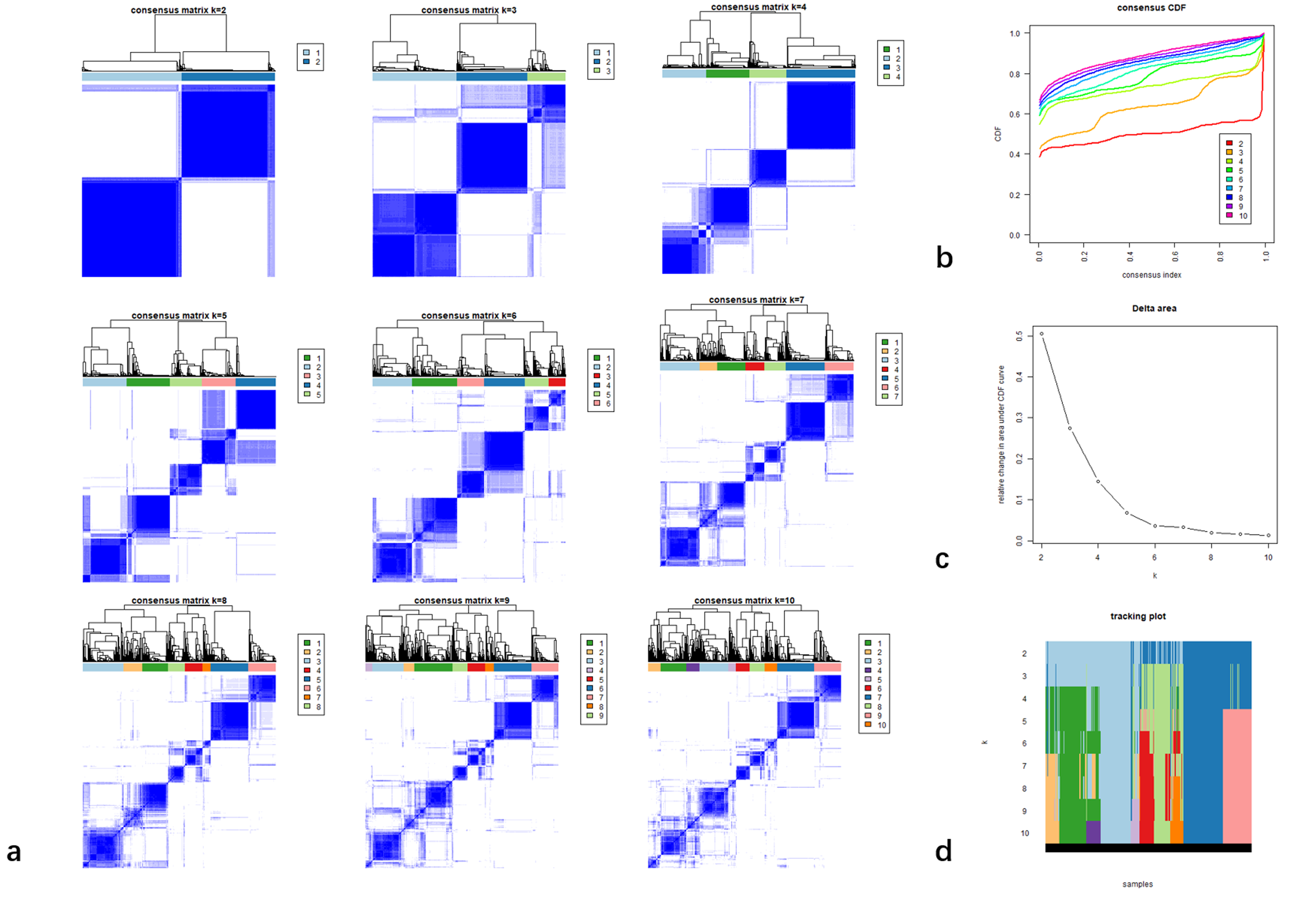

Supplement: Supplementary file 1 — Supplementary Material 1: Figure S1. Consistent cluster classification diagram. (a). Consensus matrix for k = 2 to k = 10; (b). consensus CDF curve for k = 2 to k = 10; (c). Relative change in area under CDF curve for k = 2 to k = 10; (d). Tracking plot for k = 2 to k = 10. [file 12885_2025_14176_MOESM1_ESM.tif]

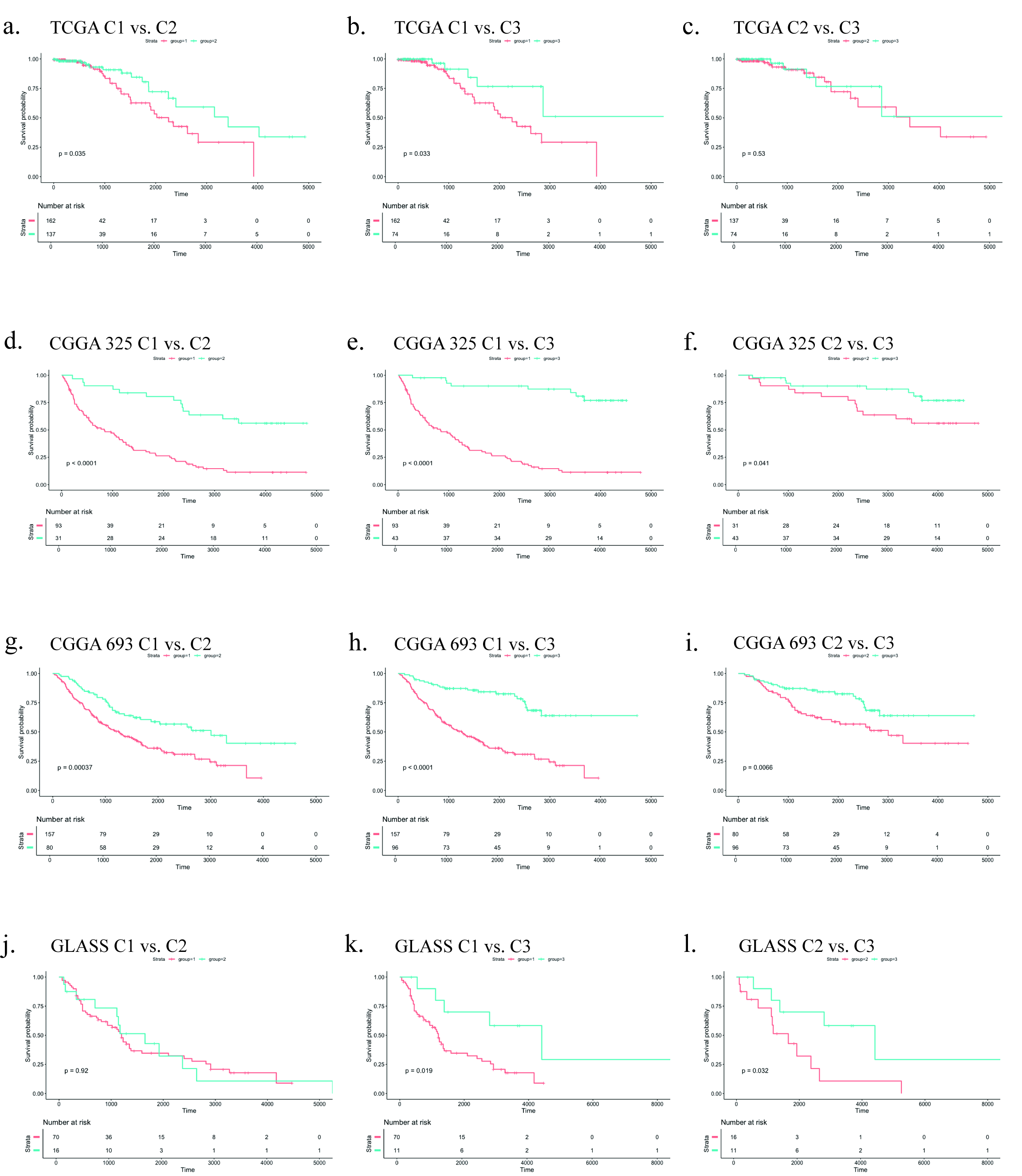

Supplement: Supplementary file 2 — Supplementary Material 2: Figure S2. The pairwise survival differences analysis in the discovery set and validation sets. (a)-(c). TCGA OS analysis; (d)-(f). CGGA 325 OS analysis; (g)-(i). CGGA 693 OS analysis; (j)-(l). GLASS OS analysis. [file 12885_2025_14176_MOESM2_ESM.tif]

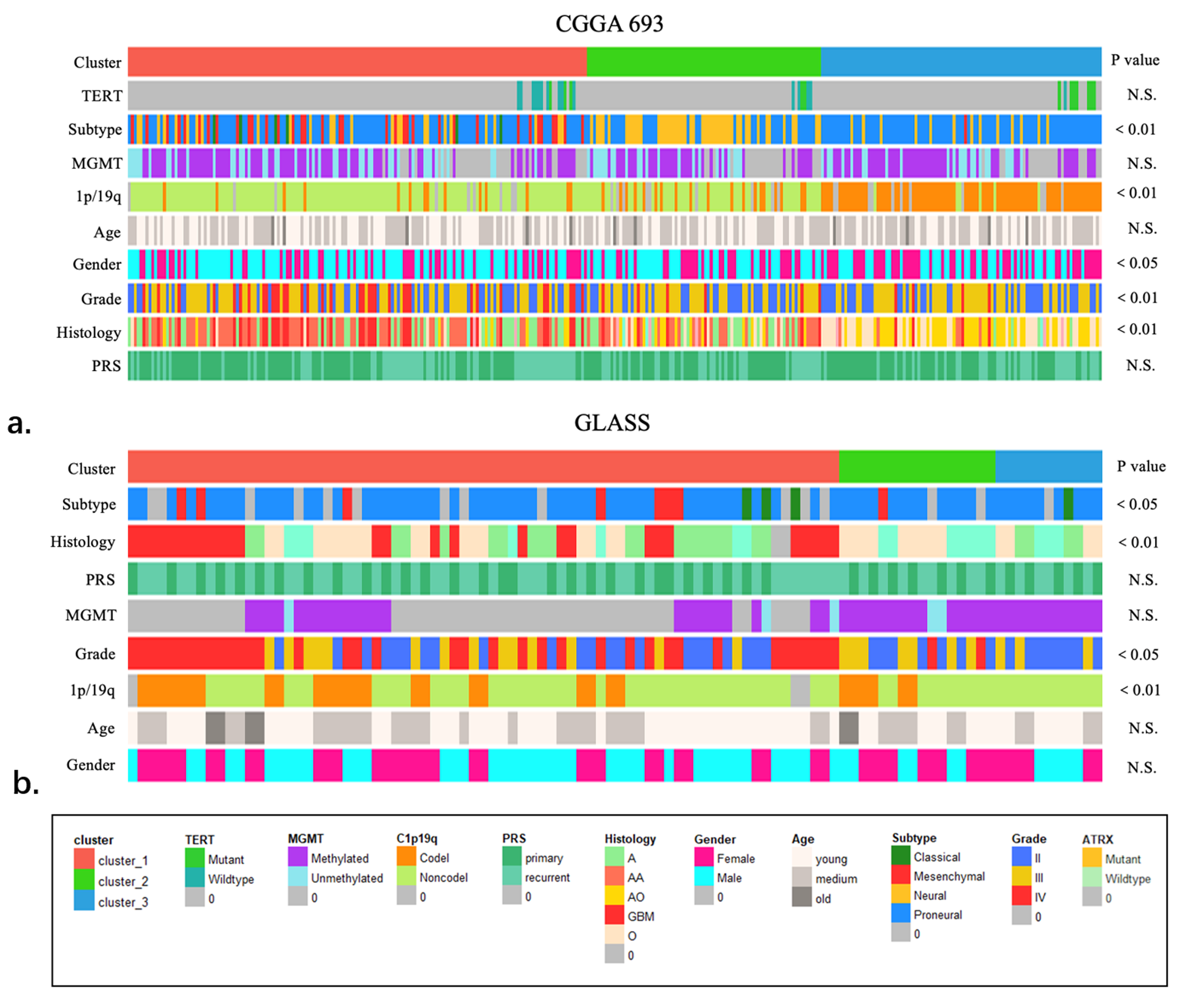

Supplement: Supplementary file 3 — Supplementary Material 3: Figure S3. Clinical characteristics of metabolic subtypes in CGGA- 693 and GLASS cohorts. (a). Clinical characteristics in the CGGA- 693 cohort; (b). Clinical characteristics in the GLASS cohort. The chi-square test was used for statistical analysis. 0 means not appliable. [file 12885_2025_14176_MOESM3_ESM.tif]

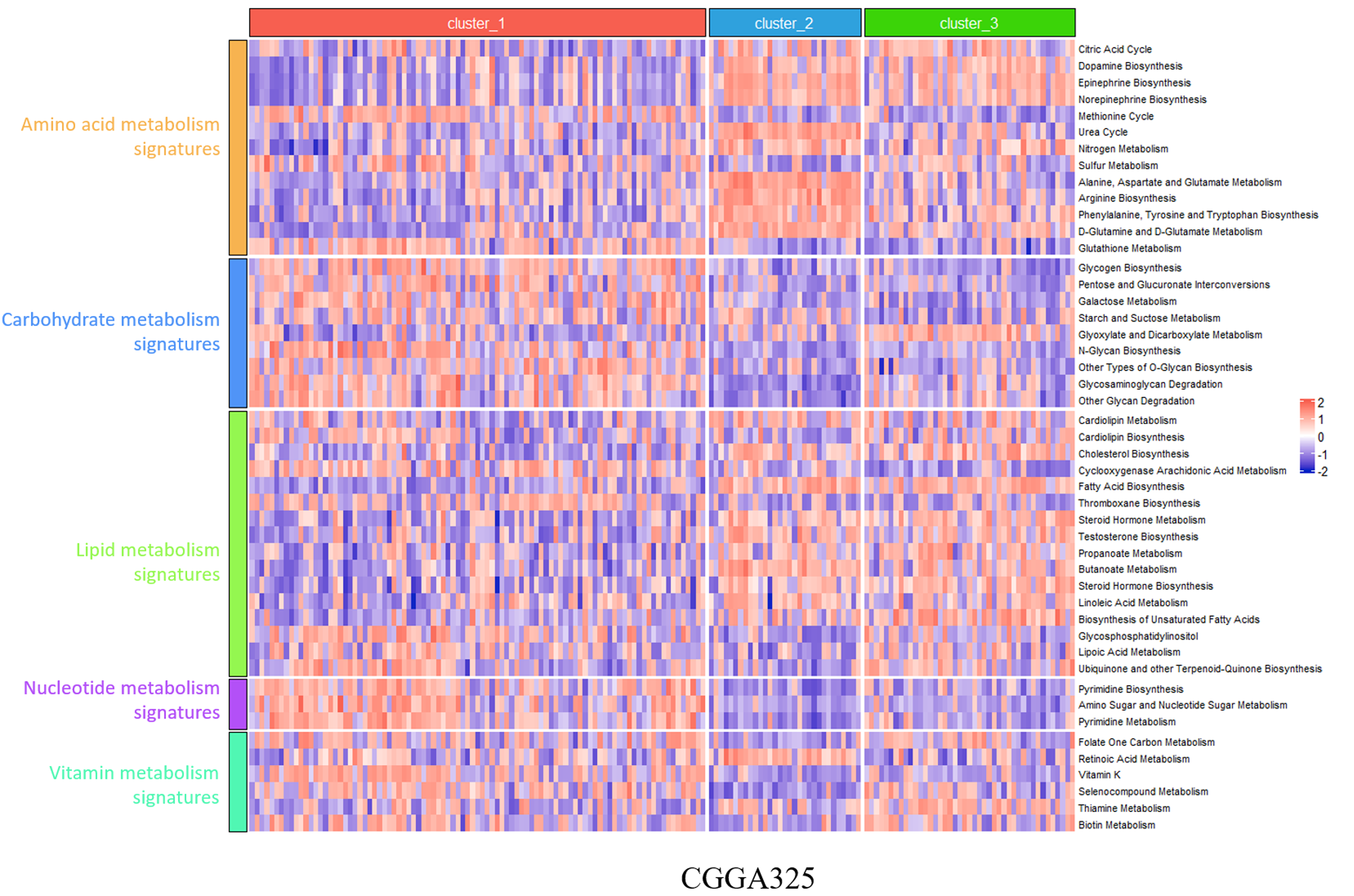

Supplement: Supplementary file 4 — Supplementary Material 4: Figure S4. Association between metabolism-relevant signatures and novel metabolic subtypes. Heatmaps of differential enrichments of metabolism-related signatures in the CGGA- 325 cohort. Amino acid, carbohydrate, lipid, nucleotide, and vitamin metabolism signatures were presented. The statistical difference was compared through the ANOVA test, and the P value < 0.05 was considered as significant. [file 12885_2025_14176_MOESM4_ESM.tif]

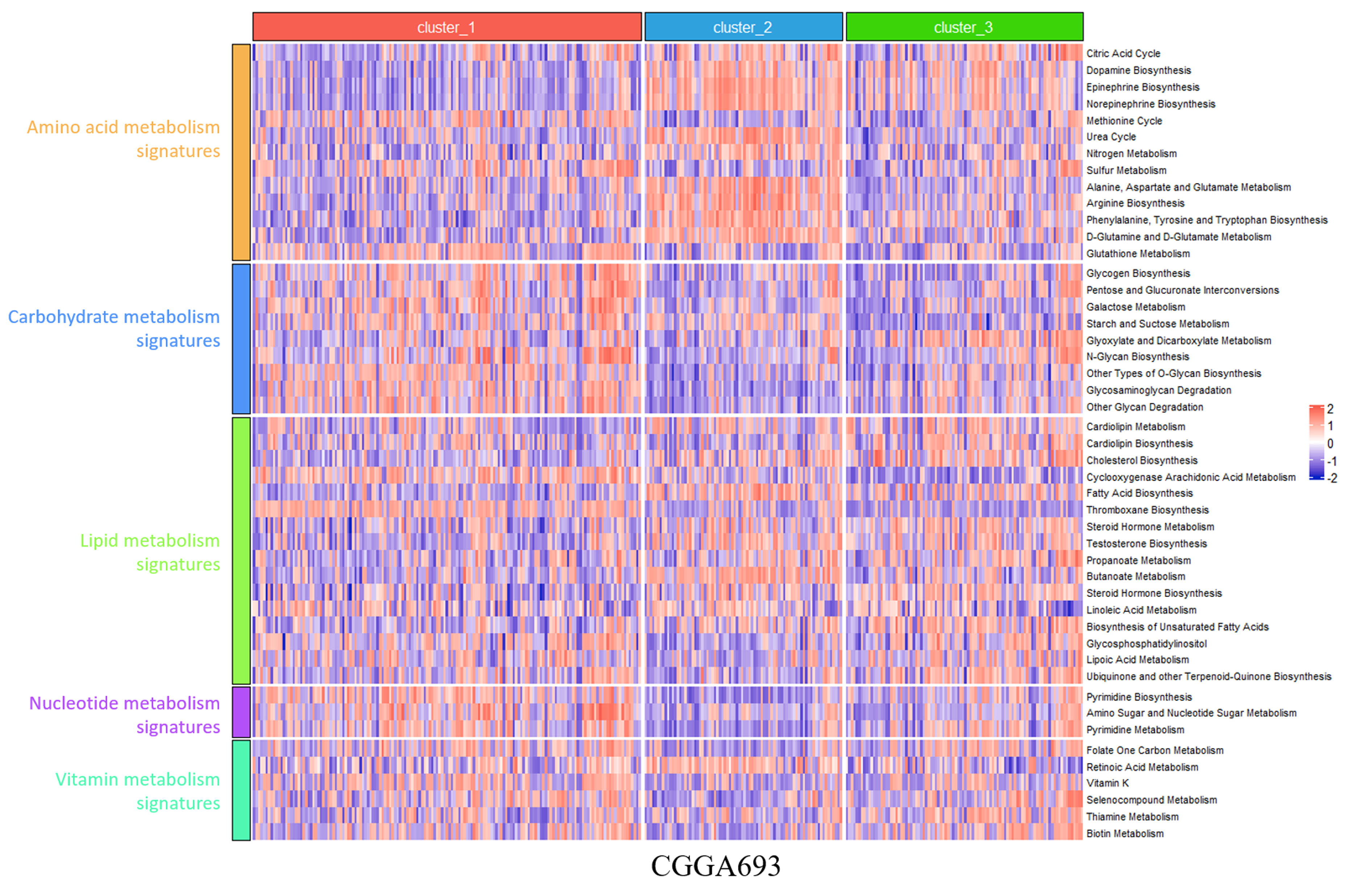

Supplement: Supplementary file 5 — Supplementary Material 5: Figure S5. Association between metabolism-relevant signatures and novel metabolic subtypes. Heatmaps of differential enrichments of metabolism-related signatures in the CGGA- 693 cohort. Amino acid, carbohydrate, lipid, nucleotide, and vitamin metabolism signatures were presented. The statistical difference was compared through the ANOVA test, and the P value < 0.05 was considered as significant. [file 12885_2025_14176_MOESM5_ESM.tif]

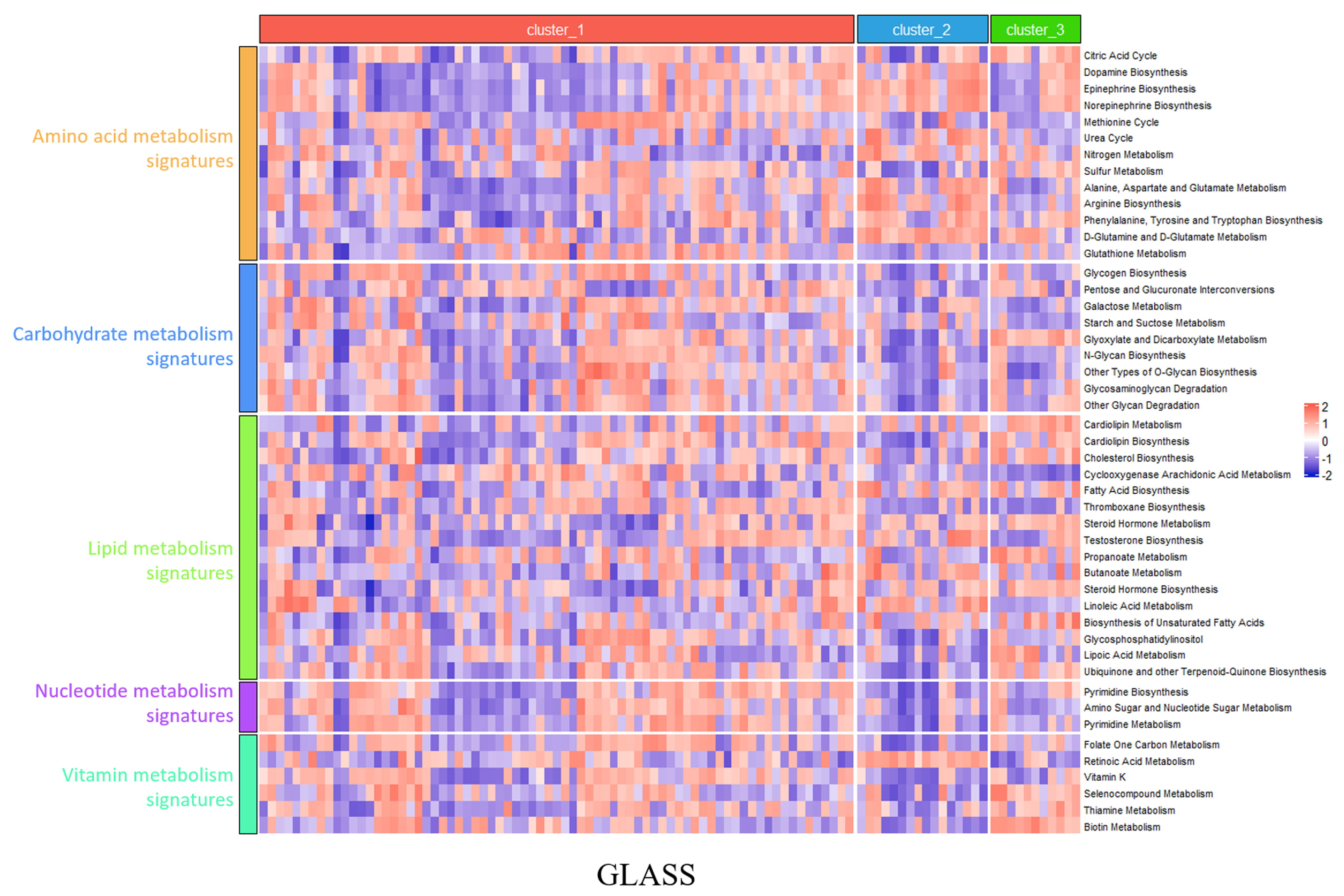

Supplement: Supplementary file 6 — Supplementary Material 6: Figure S6. Association between metabolism-relevant signatures and novel metabolic subtypes. Heatmaps of differential enrichments of metabolism-related signatures in the GLASS cohort. Amino acid, carbohydrate, lipid, nucleotide, and vitamin metabolism signatures were presented. The statistical difference was compared through the ANOVA test, and the P value < 0.05 was considered as significant. [file 12885_2025_14176_MOESM6_ESM.tif]

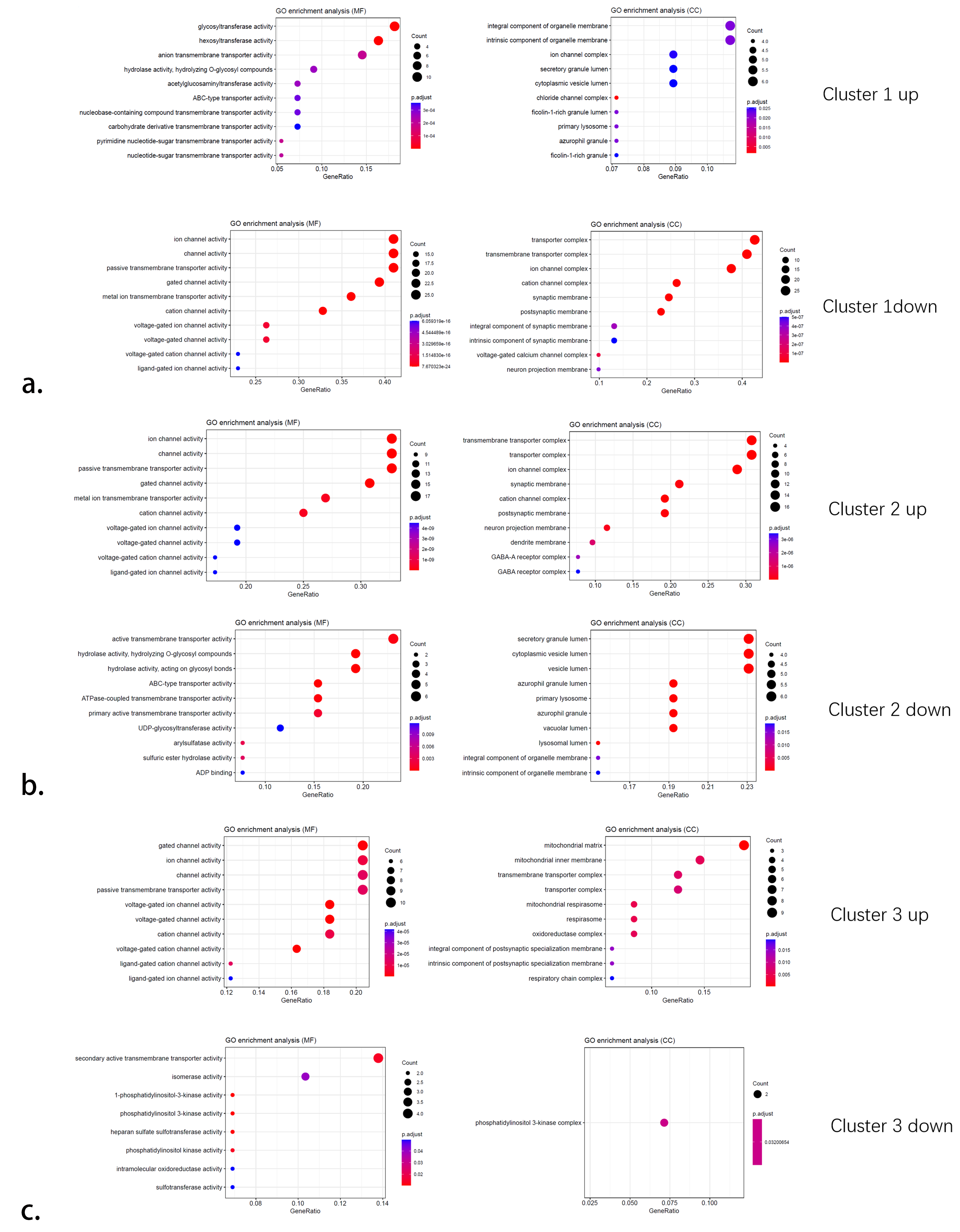

Supplement: Supplementary file 7 — Supplementary Material 7: Figure S7. Functional enrichment analysis of the metabolic subtypes in discovery and validation cohort. (a). MF and CC terms in G-O analysis of genes in cluster 1 up and down regulation; (b). MF and CC terms in G-O analysis of genes in cluster 2 up and down regulation; (c). MF and CC terms in G-O analysis of genes in cluster 3 up and down regulation. [file 12885_2025_14176_MOESM7_ESM.tif]

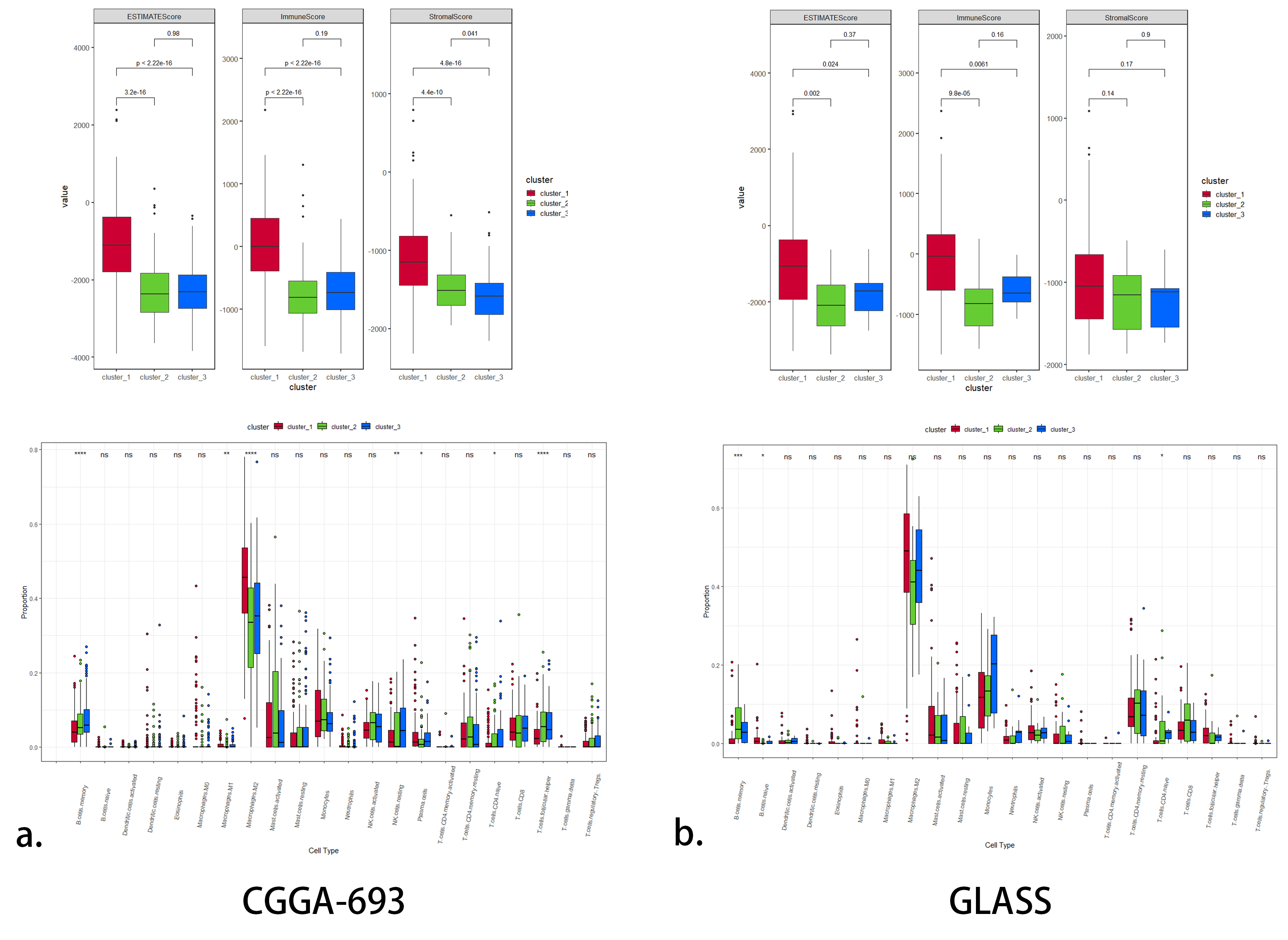

Supplement: Supplementary file 8 — Supplementary Material 8: Figure S8. Immune infiltration of three metabolic subtypes in CGGA- 693 and GLASS cohorts. (a). The ESTIMATE is used to predict immune and stromal scores and CIBERSORT is used to predict the types of immune cells in the CGGA- 693 cohort; (b). The ESTIMATE is used to predict immune and stromal scores and CIBERSORT is used to predict the types of immune cells in the GLASS cohort. [file 12885_2025_14176_MOESM8_ESM.tif]

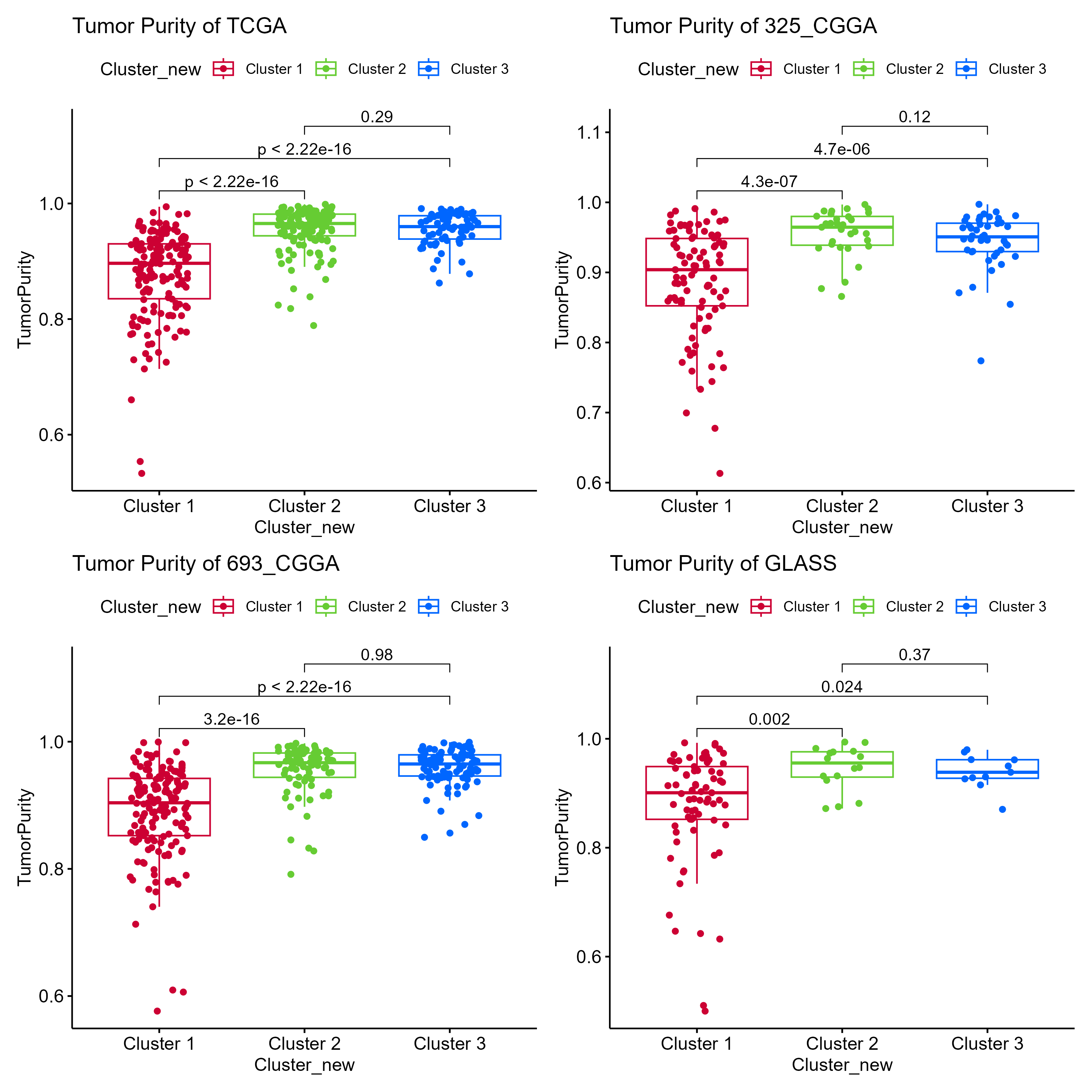

Supplement: Supplementary file 9 — Supplementary Material 9: Figure S9. Tumor purity score calculated according to the ESTIMATE algorithm in the TCGA, CGGA 325, CGGA 693, and GLASS database. (a). Tumor purity score of TCGA set; (b). Tumor purity score of CGGA 325 set; (c). Tumor purity score of CGGA 693 set; (d). Tumor purity score of GLASS set. [file 12885_2025_14176_MOESM9_ESM.tiff]

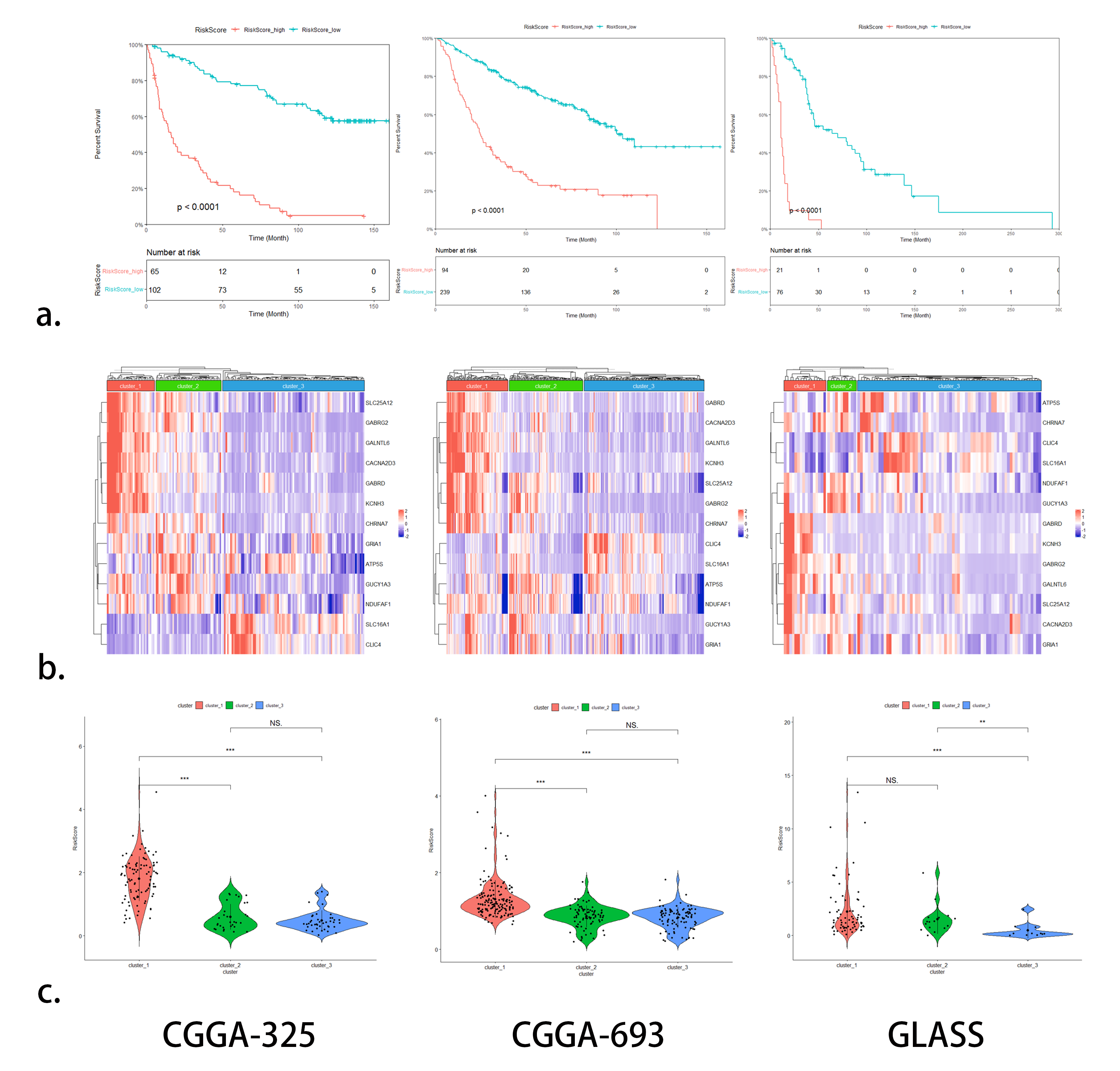

Supplement: Supplementary file 10 — Supplementary Material 10: Figure S10. Validation of the obtained metabolic signature in three validation cohorts. (a). Survival analyses of the metabolic signature in IDH-mutant gliomas. P value was calculated by the log-rank test; (b). Heatmaps show the signature gene expression of three validation cohorts; (c). Distribution of risk scores in cases stratified by metabolic subtype in three validation cohorts. [file 12885_2025_14176_MOESM10_ESM.tif]

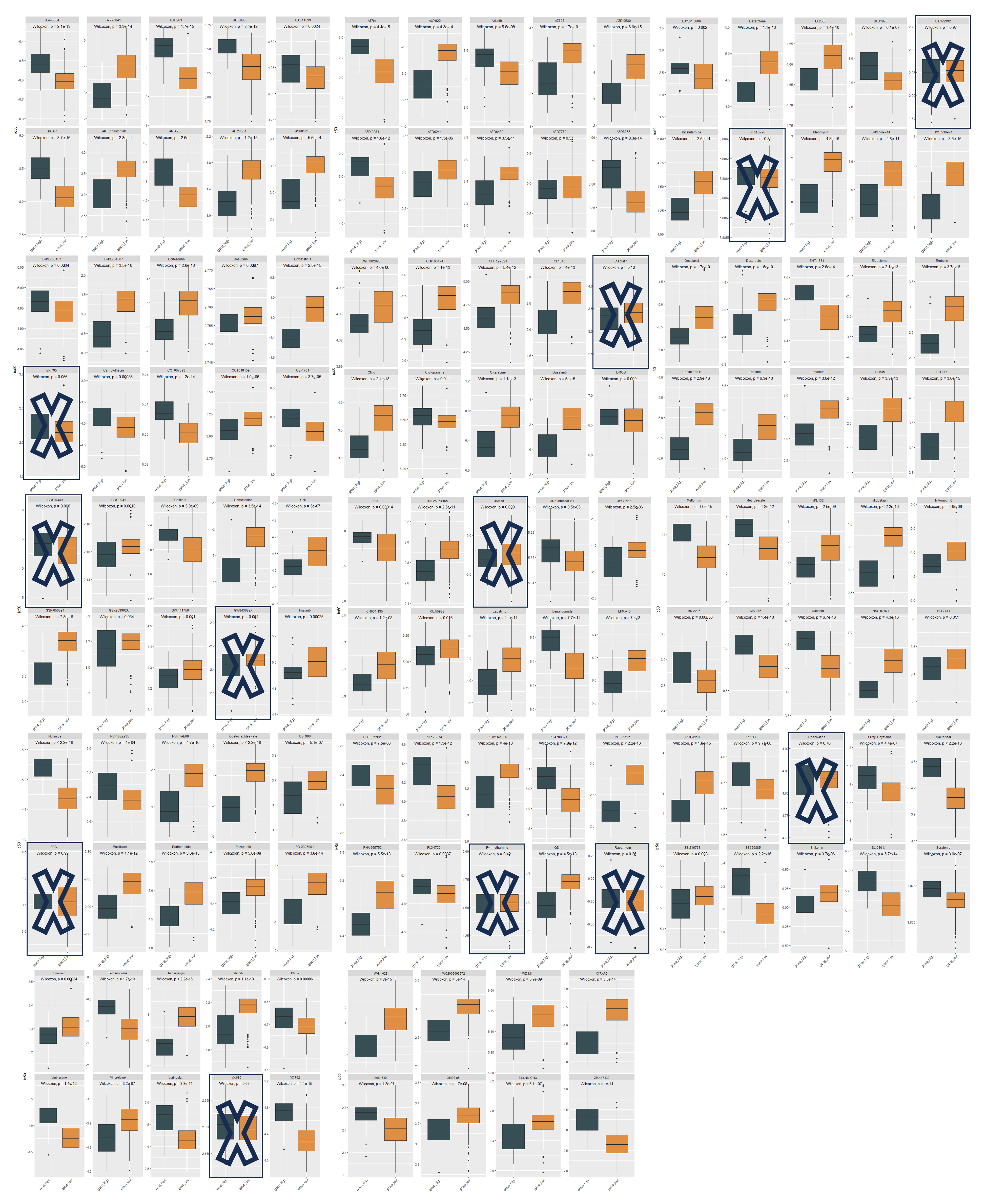

Supplement: Supplementary file 11 — Supplementary Material 11: Figure S11. Predation of drug sensitivity for the signature in CGP2014 drug library. Total 127 drugs and the resistance drug has been marked in blue cross. [file 12885_2025_14176_MOESM11_ESM.tif]
