# Supplementary material for: Novel metabolic subtypes in IDH-mutant gliomas: implications for prognosis and therapy
Source: BMC Cancer. 2025 Apr 30;25:815. doi: 10.1186/s12885-025-14176-y (PMC12044917; doi:10.1186/s12885-025-14176-y)
Supplement: Supplementary file 13 — Supplementary Material 13. Table S2. Clinical features of patients in this study. [file 12885_2025_14176_MOESM13_ESM.docx]

Table S2. Clinical characteristics of patients in this study.

| **Variable** | **TCGA** | **CGGA 325** | **CGGA 693** | **GLASS cohort** |
| --- | --- | --- | --- | --- |
|  | n=373 | n=167 | n=333 | n=100 |
| **Age** |  |  |  |  |
| <18 years | 2 | 1 | 1 | 0 |
| 18-60 years | 336 | 163 | 321 | 94 |
| > 60 years | 35 | 3 | 11 | 6 |
| **Gender** |  |  |  |  |
| Female | 162 | 65 | 142 | 48 |
| Male | 211 | 102 | 191 | 52 |
| **1P/19Q** |  |  |  |  |
| Codeleted | 149 | 59 | 112 | 29 |
| Non-codeleted | 223 | 105 | 192 | 68 |
| NA | 1 | 3 | 29 | 3 |
| **MGMT promoter** |  |  |  |  |
| Methylated | 345 | 104 | 166 | 48 |
| Unmethylated | 25 | 50 | 89 | 5 |
| NA | 3 | 13 | 78 | 47 |
| **TERT promoter** |  |  |  |  |
| Mutant | 91 | 41 | 12 | unavailable |
| WT | 141 | 91 | 15 | unavailable. |
| NA | 141 | 35 | 306 | unavailable. |
| **Grade** |  |  |  |  |
| II | 193 | 85 | 122 | 37 |
| III | 171 | 42 | 166 | 24 |
| IV | 9 | 40 | 45 | 39 |
| **Histology** |  |  |  |  |
| Astrocytoma | 115 | 71 | 160 | 24 |
| Oligoastrocytoma | 68 | 0 | 12 | 17 |
| Oligodendroglioma | 181 | 56 | 116 | 30 |
| Glioblastoma | 9 | 39 | 45 | 27 |
| NA | 0 | 1 | 0 | 2 |
| **Transcriptome subtype** |  |  |  |  |
| CL | 3 | 14 | 6 | unavailable |
| ME | 4 | 9 | 36 | unavailable |
| NE | 21 | 56 | 85 | unavailable. |
| PN | 343 | 88 | 206 | unavailable |
| NA | 2 | 0 | 0 | unavailable. |
| **PRS** |  |  |  |  |
| Primary | unavailable | 112 | 197 | 41 |
| Recurrent | unavailable. | 54 | 136 | 59 |
| NA | unavailable. | 1 | 0 | 0 |

ME: mesenchymal, NE: neural, CL: classical, PN: Proneural, PRS: Primary/Recurrent status.
