# Supplementary material for: Novel metabolic subtypes in IDH-mutant gliomas: implications for prognosis and therapy
Source: BMC Cancer. 2025 Apr 30;25:815. doi: 10.1186/s12885-025-14176-y (PMC12044917; doi:10.1186/s12885-025-14176-y)
Supplement: Supplementary file 14 — Supplementary Material 14. Table S3. Clinical features of patients in TCGA cohort. [file 12885_2025_14176_MOESM14_ESM.docx]

Table S3. Clinical characteristics of patients with distinct metabolic subtypes in TCGA cohort.

| **Variable** | **C1** | **C2** | **C3** | ***P* value** |
| --- | --- | --- | --- | --- |
|  | n=162 | n=137 | n=74 |  |
| **Age** |  |  |  | p=0.054 |
| <18 years | 0 | 2 | 0 |  |
| 18-60 years | 152 | 116 | 68 |  |
| > 60 years | 10 | 19 | 6 |  |
| **Gender** |  |  |  | p=0.270 |
| Female | 65 | 67 | 30 |  |
| Male | 97 | 70 | 44 |  |
| **1P/19Q** |  |  |  | p<0.001 |
| Codeleted | 11 | 69 | 69 |  |
| Non-codeleted | 150 | 68 | 5 |  |
| NA | 1 | 0 | 0 |  |
| **MGMT promoter** |  |  |  | p=0.008 |
| Methylated | 143 | 129 | 73 |  |
| Unmethylated | 18 | 6 | 1 |  |
| NA | 1 | 2 | 0 |  |
| **TERT promoter** |  |  |  | p<0.001 |
| Mutant | 11 | 46 | 34 |  |
| WT | 90 | 47 | 4 |  |
| NA | 61 | 44 | 36 |  |
| **Grade** |  |  |  | p=0.007 |
| II | 76 | 80 | 37 |  |
| III | 77 | 57 | 37 |  |
| IV | 9 | 0 | 0 |  |
| **Histology** |  |  |  | p<0.001 |
| Astrocytoma | 82 | 29 | 4 |  |
| Oligoastrocytoma | 41 | 25 | 2 |  |
| Oligodendroglioma | 30 | 83 | 68 |  |
| Glioblastoma | 9 | 0 | 0 |  |
| **Transcriptome subtype** |  |  |  | p<0.001 |
| CL | 3 | 0 | 0 |  |
| ME | 4 | 0 | 0 |  |
| NE | 2 | 19 | 0 |  |
| PN | 153 | 116 | 74 |  |
| NA | 0 | 2 | 0 |  |

ME: mesenchymal, NE: neural, CL: classical, PN: Proneural.
