# Supplementary material for: Novel metabolic subtypes in IDH-mutant gliomas: implications for prognosis and therapy
Source: BMC Cancer. 2025 Apr 30;25:815. doi: 10.1186/s12885-025-14176-y (PMC12044917; doi:10.1186/s12885-025-14176-y)
Supplement: Supplementary file 15 — Supplementary Material 15. Table S4. Clinical features of patients in CGGA 325 cohort. [file 12885_2025_14176_MOESM15_ESM.docx]

Table S4. Clinical characteristics of patients with distinct metabolic subtypes in CGGA 325 cohort.

| **Variable** | **C1** | **C2** | **C3** | ***P* value** |
| --- | --- | --- | --- | --- |
|  | n=93 | n=31 | n=43 |  |
| **Age** |  |  |  | p=0.909 |
| <18 years | 1 | 0 | 0 |  |
| 18-60 years | 90 | 31 | 42 |  |
| > 60 years | 2 | 0 | 1 |  |
| **Gender** |  |  |  | p=0.615 |
| Female | 38 | 13 | 14 |  |
| Male | 55 | 18 | 29 |  |
| **1P/19Q** |  |  |  | p<0.001 |
| Codeleted | 9 | 13 | 37 |  |
| Non-codeleted | 83 | 16 | 6 |  |
| NA | 1 | 2 | 0 |  |
| **MGMT promoter** |  |  |  | p=0.01 |
| Methylated | 65 | 13 | 26 |  |
| Unmethylated | 21 | 16 | 13 |  |
| NA | 7 | 2 | 4 |  |
| **TERT promoter** |  |  |  | p=0.029 |
| Mutant | 31 | 4 | 6 |  |
| WT | 46 | 17 | 28 |  |
| NA | 16 | 10 | 9 |  |
| **Grade** |  |  |  | p<0.001 |
| II | 32 | 24 | 29 |  |
| III | 26 | 4 | 12 |  |
| IV | 35 | 3 | 2 |  |
| **Histology** |  |  |  | p<0.001 |
| Astrocytoma | 51 | 14 | 6 |  |
| Oligoastrocytoma | 0 | 0 | 0 |  |
| Oligodendroglioma | 7 | 14 | 35 |  |
| Glioblastoma | 35 | 3 | 2 |  |
| NA | 1 | 0 | 0 |  |
| **Transcriptome subtype** |  |  |  | p<0.001 |
| CL | 14 | 0 | 0 |  |
| ME | 9 | 0 | 0 |  |
| NE | 13 | 26 | 17 |  |
| PN | 57 | 5 | 26 |  |
| **PRS** |  |  |  | p<0.001 |
| Primary | 49 | 27 | 36 |  |
| Recurrent | 43 | 4 | 7 |  |
| NA | 1 | 0 | 0 |  |

ME: mesenchymal, NE: neural, CL: classical, PN: Proneural, PRS: Primary/Recurrent status.
