# Supplementary material for: Novel metabolic subtypes in IDH-mutant gliomas: implications for prognosis and therapy
Source: BMC Cancer. 2025 Apr 30;25:815. doi: 10.1186/s12885-025-14176-y (PMC12044917; doi:10.1186/s12885-025-14176-y)
Supplement: Supplementary file 16 — Supplementary Material 16. Table S5. Clinical features of patients in CGGA 693 cohort. [file 12885_2025_14176_MOESM16_ESM.docx]

Table S5. Clinical characteristics of patients with distinct metabolic subtypes in CGGA 693 cohort.

| **Variable** | **C1** | **C2** | **C3** | ***P* value** |
| --- | --- | --- | --- | --- |
|  | n=157 | n=80 | n=96 |  |
| **Age** |  |  |  | p=0.931 |
| <18 years | 1 | 0 | 0 |  |
| 18-60 years | 151 | 78 | 92 |  |
| > 60 years | 5 | 2 | 4 |  |
| **Gender** |  |  |  | p=0.024 |
| Female | 58 | 32 | 52 |  |
| Male | 99 | 48 | 44 |  |
| **1P/19Q** |  |  |  | p<0.001 |
| Codeleted | 14 | 19 | 79 |  |
| Non-codeleted | 134 | 52 | 6 |  |
| NA | 9 | 9 | 11 |  |
| **MGMT promoter** |  |  |  | p=0.551 |
| Methylated | 78 | 35 | 53 |  |
| Unmethylated | 44 | 22 | 23 |  |
| NA | 35 | 23 | 20 |  |
| **TERT promoter** |  |  |  | p=0.559 |
| Mutant | 4 | 6 | 2 |  |
| WT | 5 | 5 | 5 |  |
| NA | 148 | 69 | 89 |  |
| **Grade** |  |  |  | p<0.001 |
| II | 50 | 31 | 41 |  |
| III | 72 | 43 | 51 |  |
| IV | 35 | 6 | 4 |  |
| **Histology** |  |  |  | p<0.001 |
| Astrocytoma | 105 | 46 | 9 |  |
| Oligoastrocytoma | 3 | 5 | 4 |  |
| Oligodendroglioma | 14 | 23 | 79 |  |
| Glioblastoma | 35 | 6 | 4 |  |
| **Transcriptome subtype** |  |  |  | p<0.001 |
| CL | 6 | 0 | 0 |  |
| ME | 35 | 0 | 1 |  |
| NE | 26 | 46 | 13 |  |
| PN | 90 | 34 | 82 |  |
| **PRS** |  |  |  | p=0.587 |
| Primary | 90 | 46 | 61 |  |
| Recurrent | 67 | 34 | 35 |  |

ME: mesenchymal, NE: neural, CL: classical, PN: Proneural, PRS: Primary/Recurrent status.
