# Supplementary material for: Novel metabolic subtypes in IDH-mutant gliomas: implications for prognosis and therapy
Source: BMC Cancer. 2025 Apr 30;25:815. doi: 10.1186/s12885-025-14176-y (PMC12044917; doi:10.1186/s12885-025-14176-y)
Supplement: Supplementary file 17 — Supplementary Material 17. Table S6. Clinical features of patients in GLASS cohort. [file 12885_2025_14176_MOESM17_ESM.docx]

Table S6. Clinical characteristics of patients with distinct metabolic subtypes in GLASS cohort.

| **Variable** | **C1** | **C2** | **C3** | ***P* value** |
| --- | --- | --- | --- | --- |
|  | n=73 | n=16 | n=11 |  |
| **Age** |  |  |  | p=0.638 |
| <18 years | 0 | 0 | 0 |  |
| 18-60 years | 67 | 16 | 11 |  |
| > 60 years | 6 | 0 | 0 |  |
| **Gender** |  |  |  | p=0.722 |
| Female | 33 | 9 | 6 |  |
| Male | 40 | 7 | 5 |  |
| **1P/19Q** |  |  |  | p<0.001 |
| Codeleted | 14 | 4 | 11 |  |
| Non-codeleted | 56 | 12 | 0 |  |
| NA | 3 | 0 | 0 |  |
| **MGMT promoter** |  |  |  | p=0.281 |
| Methylated | 35 | 7 | 6 |  |
| Unmethylated | 3 | 2 | 0 |  |
| NA | 35 | 7 | 5 |  |
| **Grade** |  |  |  | p=0.015 |
| II | 23 | 8 | 6 |  |
| III | 17 | 2 | 5 |  |
| IV | 33 | 6 | 0 |  |
| **Histology** |  |  |  | p<0.001 |
| Astrocytoma | 19 | 5 | 0 |  |
| Oligoastrocytoma | 14 | 3 | 0 |  |
| Oligodendroglioma | 15 | 4 | 11 |  |
| Glioblastoma | 23 | 4 | 0 |  |
| NA | 2 | 0 | 0 |  |
| **PRS** |  |  |  | p=0.288 |
| Primary | 28 | 6 | 7 |  |
| Recurrent | 45 | 10 | 4 |  |
| NA | 0 | 0 | 0 |  |

PRS: Primary/Recurrent status.
