# Supplementary material for: Novel metabolic subtypes in IDH-mutant gliomas: implications for prognosis and therapy
Source: BMC Cancer. 2025 Apr 30;25:815. doi: 10.1186/s12885-025-14176-y (PMC12044917; doi:10.1186/s12885-025-14176-y)
Supplement: Supplementary file 18 — Supplementary Material 18. Table S7. The 114 GSVA pathways. [file 12885_2025_14176_MOESM18_ESM.docx]

Table S7. Differential analysis of metabolic enrichments among three groups

|  | **P value** | | | |
| --- | --- | --- | --- | --- |
| **Metabolism-relevant signature** | TCGA | 325 | 693 | GLASS |
| ADP-Ribosylation | *** | *** | *** | ns |
| Alanine, Aspartate and Glutamate Metabolism | *** | *** | *** | * |
| Aldosterone Biosynthesis | *** | ns | ns | ns |
| alpha-Linoleic Acid Metabolism | ns | * | ** | ns |
| Amino Sugar and Nucleotide Sugar Metabolism | *** | *** | *** | * |
| Arachidonic Acid Metabolism | * | ns | ** | ns |
| Arginine and Proline Metabolism | *** | *** | *** | ns |
| Arginine Biosynthesis | *** | *** | *** | *** |
| Ascorbate and Aldrate Metabolism | ns | * | ns | ns |
| Beta-Alanine Metabolism | * | * | ** | ns |
| Biosynthesis of Unsaturated Fatty Acids | *** | *** | *** | * |
| Biotin Metabolism | *** | ** | *** | *** |
| Butanoate Metabolism | *** | *** | *** | * |
| Caffiene Metabolism | * | ns | ns | ns |
| Cardiolipin Biosynthesis | *** | * | *** | ** |
| Cardiolipin Metabolism | *** | ** | *** | ** |
| Cholesterol Biosynthesis | *** | *** | *** | * |
| Citric Acid Cycle | *** | ** | *** | * |
| Cortisol Biosynthesis | ** | *** | ns | ns |
| Cyclooxygenase Arachidonic Acid Metabolism | *** | *** | *** | *** |
| Cysteine and Methionine Metabolism | *** | ns | *** | ns |
| D-Glutamine and D-Glutamate Metabolism | *** | *** | *** | *** |
| Dopamine Biosynthesis | *** | *** | *** | * |
| Drug Metabolism by Cytochrome P450 | * | ns | ** | ns |
| Drug Metabolism by other enzymes | *** | ** | ** | ns |
| Epinephrine Biosynthesis | *** | *** | *** | *** |
| Estradiol Biosynthesis | *** | *** | ns | ns |
| Ether Lipid Metabolism | *** | * | *** | ns |
| Fatty Acid Biosynthesis | *** | *** | *** | ** |
| Fatty Acid Degradation | ns | ns | ns | ns |
| Fatty Acid Elongation | *** | *** | *** | ns |
| Folate biosynthesis | *** | ** | ns | ns |
| Folate One Carbon Metabolism | *** | *** | *** | ** |
| Fructose and Mannose Metabolism | ** | ** | ** | ns |
| Galactose Metabolism | *** | *** | *** | * |
| Gluconeogenesis | ** | ns | ** | ns |
| Glutathione Metabolism | *** | *** | *** | ** |
| Glycerolipid Metabolism | *** | *** | *** | ns |
| Glycerophospholipid Metabolism | *** | *** | *** | ns |
| Glycine, Serine and Threonine Metabolism | ** | *** | ns | ns |
| Glycogen Biosynthesis | *** | *** | *** | * |
| Glycogen Degradation | ns | ns | ns | ns |
| Glycolysis | * | ns | ** | ns |
| Glycosaminoglycan Biosynthesis | *** | *** | *** | ns |
| Glycosaminoglycan Degradation | *** | *** | *** | ** |
| Glycosphingolipid Biosynthesis | *** | *** | *** | ns |
| Glycosphosphatidylinositol | *** | *** | *** | ** |
| Glyoxylate and Dicarboxylate Metabolism | *** | *** | *** | *** |
| Heme Biosynthesis | ns | ns | ns | ns |
| Hexosamine Biosynthesis | ** | * | * | ns |
| Histidine Metabolism | ns | * | * | ns |
| Homocysteine Biosynthesis | *** | *** | *** | ns |
| Inositol Phosphate Metabolism | *** | *** | *** | ns |
| Ketone Biosynthesis and Metabolism | *** | *** | *** | ns |
| Kynurenine Metabolism | *** | *** | *** | ns |
| Linoleic Acid Metabolism | ** | ** | * | ** |
| Lipoic Acid Metabolism | *** | * | ** | * |
| Lysine Degradation | *** | ** | *** | ns |
| Metabolism of Xenobiotics by Cytochrome P450 | * | ns | * | ns |
| Methionine Cycle | *** | *** | *** | * |
| Mucin Type O-Glycan Biosynthesis | *** | *** | *** | ns |
| Neomycin, Kanamysin and Gentamicin Biosynthesis | * | * | ** | ns |
| N-Glycan Biosynthesis | *** | *** | *** | ** |
| Nicotinamide Adenine Dinucleotide Biosynthesis | * | ns | * | ** |
| Nicotinamide Adenine Metabolism | *** | *** | *** | ns |
| Nicotinate and Nicotinamide Metabolism | *** | ns | ns | ns |
| Nitrogen Metabolism | *** | *** | *** | ** |
| Norepinephrine Biosynthesis | *** | *** | *** | *** |
| Other Glycan Degradation | *** | *** | *** | * |
| Other Types of O-Glycan Biosynthesis | *** | *** | *** | * |
| Oxidative Phosphorylation | ** | * | ns | ** |
| Pantothenate and CoA Biosynthesis | *** | *** | *** | ns |
| Pentose and Glucuronate Interconversions | *** | *** | *** | * |
| Pentose Phosphate | ** | ns | * | ns |
| Phenylalanine Metabolism | *** | ** | *** | ns |
| Phenylalanine, Tyrosine and Tryptophan Biosynthesis | *** | *** | *** | * |
| Polyamine Biosynthesis | *** | *** | *** | ns |
| Porphyrin and Chlorophyll Metabolism | ** | * | ns | * |
| Primary Bile Acid Biosynthesis | *** | ns | *** | ns |
| Propanoate Metabolism | * | *** | *** | ** |
| Prostaglandin Biosynthesis | ns | ns | ns | ns |
| Prostanoid Biosynthesis | ** | ns | ns | ns |
| Purine Biosynthesis | ns | ns | ns | * |
| Purine Metabolism | * | ** | ns | ns |
| Pyrimidine Biosynthesis | *** | *** | *** | ** |
| Pyrimidine Metabolism | *** | *** | *** | ** |
| Pyruvate Metabolism | *** | *** | *** | ns |
| Remethylation | ns | ns | * | ns |
| Retinoic Acid Metabolism | *** | *** | *** | *** |
| Retinoid Metabolism | *** | * | *** | ns |
| Retinol Metabolism | ns | ns | ns | ns |
| Riboflavin Metabolism | *** | *** | *** | ns |
| Selenocompound Metabolism | *** | *** | *** | ** |
| Shingolipid Metabolism | *** | ns | ns | ns |
| Sirtuin Nicotinamide Metabolism | *** | * | ** | ns |
| Starch and Suctose Metabolism | *** | ** | *** | * |
| Steroid Biosynthesis | *** | *** | *** | ns |
| Steroid Hormone Biosynthesis | *** | *** | *** | * |
| Steroid Hormone Metabolism | *** | *** | *** | * |
| Sulfur Metabolism | *** | ** | *** | ** |
| Taurine and Hypotaurine Metabolism | *** | *** | *** | ns |
| Terpenoid Backbone Biosynthesis | *** | ns | ** | ns |
| Testosterone Biosynthesis | *** | *** | *** | * |
| Thiamine Metabolism | *** | *** | ** | * |
| Thromboxane Biosynthesis | *** | *** | *** | *** |
| Transsulfuration | * | ns | ns | ns |
| Tryptophan Metabolism | ns | *** | ns | * |
| Tyrosine Metabolism | *** | * | *** | ns |
| Ubiquinone and other Terpenoid-Quinone Biosynthesis | *** | ** | *** | *** |
| Urea Cycle | *** | *** | *** | * |
| Valine, Leucine and Isoleucine Biosynthesis | * | ns | ns | ns |
| Valine, Leucine and Isoleucine Degradation | ns | ns | ns | ns |
| Vitamin B6 Metabolism | *** | *** | *** | ns |
| Vitamin K | *** | *** | *** | ** |

ns, *P*>0.05; *, *P≤*0.05; **, *P*<0.01; ***, *P*<0.001.
